# PSMC6 regulation of ovarian cancer cisplatin resistance unravels a new mode for proteasome targeting

**DOI:** 10.7150/ijbs.104612

**Published:** 2025-02-26

**Authors:** Matteo Costantino, Luca Mirra, Padraig D'arcy, Cristina Corno, Nives Carenini, Elisabetta Corna, Johannes Gubat, Chiara M. Ciniselli, Pietro Pratesi, Paolo Verderio, Stig Linder, Giovanni L. Beretta, Paola Perego

**Affiliations:** 1 Unit of Molecular Pharmacology, Department of Experimental Oncology, Fondazione IRCCS Istituto Nazionale dei Tumori, Milan, Italy.; 2 Department of Biomedical and Clinical Sciences, Linköping University, Sweden.; 3 Unit of Bioinformatics and Biostatistics, Department of Epidemiology and Data Science, Fondazione IRCCS Istituto Nazionale dei Tumori, Via Venezian, 1, 20133 Milan, Italy.; 4 Division of Biochemistry, Department of Medical Biochemistry and Biophysics, Karolinska Institutet, Stockholm, Sweden.

**Keywords:** ovarian carcinoma, PSMC6, proteasome, cisplatin, drug resistance, DUSP6

## Abstract

Ovarian carcinoma has still a poor prognosis. CRISPR/Cas9 loss-of-function screen revealed a relationship between the PSMC6 proteasome subunit expression and survival of cisplatin-sensitive and -resistant ovarian carcinoma cells. Increased levels of PSMC6 were evidenced in multiple ovarian carcinoma cell lines versus normal cells. An association between PSMC6 levels and tumour stages as well as with a reduced progression-free survival was found. Since a PSMC6 interactome analysis evidenced limited knowledge on PSMC6 biology, mechanistic studies were carried out. PSMC6 knockdown indicated reduced cell growth and clonogenicity in cisplatin-sensitive IGROV-1 and -resistant IGROV-1/Pt1 cells, with a higher impact in resistant cells. This behaviour was accompanied by the accumulation of ubiquitinated proteins and down-regulation of ERK1/2 phosphorylation mediated by increased DUSP6. PSMC6 silencing increased sensitivity to cisplatin in IGROV-1/Pt1 cells as shown by clonogenic assay and 3D spheroids. Since PSMC6 knockdown did not change sensitivity to 20S and 19S proteasome inhibitors, we suggest a new mode of proteasome targeting by interference with a proteasome ATPase. Overall, a link between PSMC6 and ovarian carcinoma aggressiveness is envisioned, highlighting PSMC6 as a potential diagnostic and therapeutic target.

## Introduction

Ovarian carcinoma is a heterogeneous disease responsible for the majority of deaths for gynecological cancers 1. Platinum (Pt)-based therapy still remains the standard approach for this disease with the use of cisplatin and carboplatin, the latter showing a more tolerable profile. The advances in the understanding of the molecular landscape have led to the approval of Poly-ADP-ribose polymerase (PARP) inhibitors for maintenance following response to Pt-based chemotherapy in BRCA1/2 mutant and sporadic tumors 2. Monoclonal antibody-based therapies, such as bevacizumab, are also clinically available for ovarian cancer treatment 3. Given the prevalent occurrence of drug resistance and progression through first-line platinum-based therapy in most patients, elucidating the mechanisms underlying poor response is vital.

Pt-drug resistance is a multifaceted phenomenon involving various contributors 4. Notably, cellular processes hindering drug interaction with DNA, such as reduced drug accumulation or heightened detoxification, may play pivotal roles. Thiol metabolism has been reported to operate both as an intrinsic feature of cancer cells as well as in the tumor microenvironment via the action of cancer-associated fibroblasts 5. The involvement of transporters, gated channels and passive diffusion in Pt drug accumulation, as well as the involvement of voltage-regulated anion channels has been reported 6,7,8. Restoration of DNA repair has been linked to resistance, as shown by the capability of secondary BRCA1 mutations to restore the wild-type gene reading frame in ovarian carcinomas with primary or acquired cisplatin resistance 9. Secondary BRCA1 mutations able to restore the wild-type gene reading frame and DNA repair in ovarian carcinomas have been associated to resistance to cisplatin and PARP inhibitors 10, implying the need for new target discovery. In spite of the multitude of achievements in the understanding of the molecular bases of Pt resistance, such knowledge has only in part increased the therapeutic options, with an urgent need to identify key players.

In this scenario, emerging evidence shows that deregulation of the Ubiquitin Proteasome System (UPS) may contribute to cell response to antitumor agents and to the development of drug resistance 11. The proteasome is a large multiprotein complex found in all cellular compartments, that mediates the ubiquitin-dependent proteolytic cleavage of target proteins in an ATP-dependent manner 12. These features regulate various cellular processes including protein quality control, cell cycle progression, DNA replication, transcription, modulation of signalling pathways, DNA repair, stress responses and immune responses 13. One of the executors of the UPS in the 26S proteasome, the 26S regulatory subunit 10B, is encoded by the Proteasome 26S Subunit, ATPase 6 (PSMC6) gene. PSMC6 is one of the six proteasome AAA-ATPases (Regulatory Particle Triple-A ATPases; Rpt1-6) belonging to the Regulatory Particle caps, made up of 19 integral subunits, present on one or both ends of the 20S proteasome core particle (20S CP) 14. The ATPase subunits use the energy from ATP hydrolysis to unfold the substrate protein. Once the gate of the central channel in the core particle is open, the unfolded protein is translocated into the central chamber of the 20S CP for degradation 14.

In the present study, the interplay between expression of proteasome subunits and cisplatin resistance has been investigated by a CRISPR/Cas9 dropout screen in a pair of cell lines that resemble clinically relevant features of ovarian carcinoma (i.e., activation of survival pathways and *TP53* mutation). Following lentiviral transduction with Cas9 and gRNAs against proteasome components, cisplatin-sensitive IGROV-1 and -resistant IGROV-1/Pt1 cells were sequenced to identify genes necessary for survival. Among the proteasome subunits found to be essential for the survival of IGROV-1/Pt1 cells, PSMC6 knockout produced the greatest growth inhibitory effects.

Since PSMC6 expression was enhanced in tumor versus normal cells and was found to be associated with progression-free survival, PSMC6 role was further addressed in ovarian cancer cells using a functional approach based on RNA interference in 2D and 3D preclinical models. Taken together, our results indicate that PSMC6 targeting impairs the proliferation and survival abilities of ovarian cancer cells and diminishes drug resistance through the canonical MAPK pathway. Thus, PSMC6 targeting may be an additional therapeutic strategy against the anticancer drug resistance besides inhibition of 20S or 19S proteasome functions.

## Material and methods

### Cell lines and growth conditions

A panel of human ovarian carcinoma cell lines was used for this study including pairs of cisplatin-sensitive (IGROV-1, A2780, OVCAR-5, TOV21G, TOV112D, CAOV3, ES-2, PEO1, SKOV-3) and -resistant, (IGROV-1/Pt1, A2780/CP, OVCAR-5/Pt, PEO4, TOV21G/Pt) variants. The resistant variants were obtained as previously reported 15-18 or from parental cells upon chronic exposure to increasing cisplatin concentrations (TOV21G/Pt). The TOV21G, TOV112D, CAOV3 and ES-2 cell lines were from ATCC (Rockville, MD, USA). PEO1 and PEO4 were obtained from Sigma Aldrich (St. Louis Missouri, USA). OVCAR-3, OVCAR-4 and OVCAR-8 were kindly provided by the DCTP Tumor repository (NCI, Frederick, MD, USA). Cells were cultured in plastic flasks (25 cm² or 75 cm² area) and all grown in RPMI-1640 supplemented with 10% fetal bovine serum (FBS) except than CAOV3 cells maintained DMEM plus 15% FBS and ES-2 grown in McCoys 5A plus 10% FBS, at 37°C in 5% of CO_2_. Immortalized human ovarian epithelial cells-SV40 (OSE-SV40, abm, Richmond, Canada) were cultured in complete Prigrow I medium (abm) at 37°C in 5% of CO_2_. The immortalized human fallopian tube secretory epithelial cells (FT240, abm) were cultured in complete Prigrow III medium (abm) at 37°C in 5% of CO_2_. The cells were routinely tested for mycoplasma contamination by using the MycoAlert kit (Lonza Basel, Switzerland) and their identity verified by Short Tandem Repeat analysis (ATCC, LGC standard, Milan Italy). For 3D growth, we elected to generate spheroids by seeding 10.000 cells in U-bottom 96-well plates (Thermo Scientific, Waltham, MA, USA), a condition that allow to obtain spheroids already after 24 h.

### CRISPR/Cas9 dropout screen

Stable Cas9-expressing IGROV-1 and IGROV-1/Pt1 cells were generated via lentiviral transduction with pLenti-Cas9-T2A-Blast-BFP, expressing a codon-optimized WT SpCas9 fused to a blasticidin-S-deaminase - mTagBFP fusion protein through a self-cleaving peptide. Post 2 µg/mL blasticidin selection, a stable BFP+ cell population was obtained. For the guide library, the virus titer was estimated, and Cas9-BFP-expressing target cells were transduced in duplicate at an MOI of 0.3, ensuring coverage of 1,000x (1,000 cells per guide). Puromycin selection was applied from day 2 to day 6 post-transduction, maintaining cell numbers at ≥ 80 million/replicate, with a control sample of 80 million cells per replicate harvested on day 4. Genomic DNA isolation occurred after 21 days, followed by Illumina NovaSeq sequencing. NGS data analysis encompassed the use of MaGeCK software and UMI lineage dropout analysis for a comprehensive evaluation.

### RNA Extraction and quantitative Real Time PCR analysis

Gene expression was evaluated by qRT-PCR. RNA was extracted from cells or ovarian tumor specimens with the RNeasy Plus Mini kit (Qiagen, Hilden, Germany). Following extraction, 1 µg of RNA from each sample was retrotranscribed with the High-Capacity cDNA Archive kit (Thermo Fisher Scientific) according to manufacturer's protocols. Reactions, including master mix (7.5 μL, TaqMan Universal Fast PCR Master Mix, Thermo Fisher Scientific), cDNA (3.75 μL) and the specific assay (0.75 μL) were in a 15 μL volume. Real time qRT-PCR was carried out in triplicate and run on the QuantStudio 7 Flex instruments (Thermo Fisher Scientific), and analysis was performed using QuantStudio 6 and 7 Flex software. The following TaqMan assays were used: GAPDH (Hs99999905_m1), PSMC6 (Hs01584140_m1) and RPS13 (Hs01011488_g1) 19 (Thermo Fisher Scientific). To determine relative expression levels, the relative quantification (RQ) manager software (Thermo Fisher Scientific) was used 20.

### Western blot analysis

Western blot analysis was carried out as previously described 21. Lysates were loaded on SDS-PAGE and fractionated proteins blotted on nitrocellulose membranes. Following pre-blocking in PBS containing 5% (w/v) dried no fat milk, filters were incubated overnight at 4°C with antibodies to anti-PSMC6 and anti-Ubiquitin Antibody (Abcam, Cambridge, UK), anti-PSMC5 (Bethyl Laboratories, Montgomery, Texas, USA), anti-AKT (BD Bioscences, Franklin Lakes, NJ, USA), anti-P-AKT, anti-ERK1/2 and anti-P-ERK1/2 (Cell Signaling technology, Danvers, MA, USA), anti-DUSP5 (Abnova, Taipei City, Taiwan), anti-DUSP6 (Abcam), anti cleaved caspase-3 and anti p21^cip1/waf1^ (Cell Signaling technology). An anti-β-tubulin antibody (Abcam), an anti-vinculin and an anti-actin antibody (Sigma-Aldrich) were used as control for loading. Antibody binding to blots was detected by chemoluminescence.

### Pilot study and statistical analysis

For the human study on ovarian carcinoma patients, standard descriptive statistics (medians and ranges for continuous variables and frequency tables for categorical variables) were used to describe the study sample characteristics.

The used case material was obtained from the institutional biobank which contains retrospectively collected samples. Consent for the use of clinical information for translational research was obtained for all patients. The study was approved from the review board of the Fondazione IRCCS Istituto Nazionale dei Tumori (protocol INT 23/21).

The population consists of a cohort of 134 consecutively recruited patients with a diagnosis of ovarian carcinoma. Statistical analysis was performed considering PSMC6 expression relatively to RPS13, in terms of log2RQ (i.e. -ΔCt). To assess the association between the PSMC6 levels and the main clinic-pathological characteristics, according to the underline distribution of the data, the non-parametric KW or W test were used. Correction for multiple comparisons was performed according to Bonferroni's adjustment. Univariate logistic regression models were further implemented with the estimation of the OR and corresponding 95% Confidence Interval. All statistical analyses were performed with the SAS software (version 9.4.; SAS Institute, Inc., Cary, NC, USA), adopting a nominal α level of 5%. Graphical representations were created using the R software (R Foundation for Statistical Computing, Vienna, Austria) with the ggplot2 package.

### Public database and STRING interaction network analysis

The dataset of the Cancer Genome Atlas (TCGA) Research network was used to study the relationship between PSMC6 levels and patient survival 22. In addition, an interaction network analysis was carried out using STRING, a database used to find known and predicted protein-protein interactions (https://string-db.org).

### Loss of function studies

Cells were plated in 6-well plates (24.000 cells/cm²) and, 24 h later, they were transfected using Opti-MEM transfection medium (Gibco by Thermo Fisher Scientific) and Lipofectamine RNAiMAX (Thermo Fisher Scientific), with 100 nM of small interfering RNAs (siRNAs) directed to PSMC6 after preliminary experiments to optimize the siRNA concentration. PSMC6 siRNAs were: Silencer Select siRNA 11383 (siRNA A) and siRNA 11385 (siRNA B, Thermo Fisher Scientific) and control siRNA (Silencer Select Negative Control #2 siRNA, Thermo Fisher Scientific). After 5 h, the transfection mix was replaced with complete medium and 48, 72 and 144 h later cells were harvested for analysis of knockdown efficiency by quantitative Real Time PCR (qRT-PCR) and Western blotting. When spheroids were generated, cells were harvested 5 h after transfection start and seeded in U-bottom 96 well plates (10.000 cells/well); 24 h later spheroids were exposed to 100 µM cisplatin and their diameter was measured 72 h after treatment. Results are expressed as relative diameter value obtained by dividing the measure of treated spheroids for that of the untreated one at 72 h.

### Drugs and cell sensitivity assays

Cisplatin (Sandoz S.p.A. Milan, Italy) was diluted in saline; bortezomib (Sigma-Aldrich) and bAP-15 (Selleck Chemicals LLC, Houston, TX, USA) were dissolved in DMSO at 25 and 10 mM, respectively. Drugs were directly added to culture medium to achieve the final desired concentrations. DMSO final concentration never exceeded 0.25%.

The cell growth inhibition assay was employed to assess the effect of PSMC6 knockdown on cell growth and on sensitivity to drugs (bAP-15, bortezomib). Cell sensitivity to cisplatin was evaluated by growth inhibition assay. Forty-eight hours following transfection, cells were harvested and re-seeded in 12-well plates (60.000 cells/cm^2^). Twenty-four hours later cells were exposed to different drug concentrations. After 72 h of exposure, cells were collected and counted (i.e., 144 h from transfection start) using an automatic counter (ZB1, Beckman Coulter, CA, USA). The percentage of cell growth compared to the control untreated cells was calculated. IC_50_ is defined as the drug concentration inhibiting cell growth by 50%.

The colony formation assay was used to assess the ability of transfected and untransfected cells to form colonies after cisplatin exposure. Cells were seeded in 6-well plates (untransfected and control siRNA 1.000 cells/well; siRNA A and B 5.000 cells/well) 48 h after transfection start. Twenty-four hours after seeding, cells were treated with cisplatin at different concentrations and maintained in an incubator for about 12 days. Following fixation with ethanol (96% v/v, Sigma-Aldrich, St. Louis, MO, United States) and staining with crystal violet (2% w/v in ethanol 70%), the number of colonies was counted from images obtained using Essential V6 imaging (UVITEC Cambridge, Cambridge, UK).

The clonogenic ability of silenced cells was also tested in agar. Cells were harvested 48 h after transfection start and seeded (20.000 cells) in 35 mm (9,6 cm^2^) dishes 23. Following 10 days of incubation, the colonies were stained with a 1 mg/ml solution of p-iodonitrotetrazolium violet (Sigma), and after 24 h of incubation were counted using a magnificator.

Drug sensitivity of 3D tumor cell cultures, i.e., spheroids, was assessed in U-bottom 96 well plates by exposure to 100 μM cisplatin for 72 h. A single spheroid was evident in each well 24 h after seeding 10.000 cells in U-bottom 96-well plates. Thus, 24 h after cell seeding, spheroids were exposed to cisplatin. Seventy-two h later, spheroids were observed under light microscopy (Leica DM IRB, Leica Microsystems, Buccinasco, Italy) equipped with a digital camera and diameters were measured using the Optical Preview Digital Camera software (Leica Microsystems).

### Apoptosis analyses

The Annexin V-binding assay (Immunostep, Salamanca, Spain) was used to measure basal apoptosis levels. Cells were harvested 72 h after transfection with siRNAs and, after washing with PBS, cells were processed according to manufacturer's protocol. Annexin V-binding was examined by flow cytometry (BD Accuri C6, Becton Dickinson, Milan, Italy) by acquiring ten thousand events for each sample. Instrument software (Becton Dickinson) was used to analyze the results.

### Quantitative analysis of total ERK1/2 and phospho-ERK1/2

The ELISA (AB176641, Abcam) was used to evaluate the total ERK and phospho-ERK levels upon PSMC6 silencing according to the manufacturer's protocol. Briefly, cells (240.000 cells/well) were seeded in 6-well plate and 24 h later they were silenced for 5 h with 100 nM siRNA A and siRNA B. After 72 h, cells were harvested and the levels of ERK1/2 and phospho-ERK1/2 were defined according to the manufacturer's instructions.

### Human proteome array

Human Apoptosis Array kit (R&D Systems, Abingdon, UK) was used for the analysis of the expression profiles of cell stress-related factors, according to the manufacturer's protocol. Briefly, 24 h after seeding, cells were exposed to 100 nM siRNAs directed to PSMC6 (Thermo Fisher Scientific) or control siRNA (Silencer Select Negative Control #2 siRNA, Thermo Fisher Scientific). Five hours later, transfection mix was replaced with complete medium and, 72 h later, cells were harvested and lysed. Protein extracts (300 µg) were incubated overnight onto the array. After washing, a cocktail of biotinylated detection antibodies was added to the array. Streptavidin-HRP and chemiluminescent detection reagents were then used. Spots detected on the array by exposing a film correspond to the levels of bound protein.

### Additional statistical analyses

For preclinical experiments, relationships were investigated by using the non-parametric Wilcoxon (W) or Kruskal-Wallis (KW) tests according to the number of considered groups 24. Corresponding p-values were estimated according to exact test or via Monte Carlo approaches. Correction for multiple comparisons were performed according to Bonferroni adjustment. In addition, ANOVA followed by Bonferroni's test was used. For analyses from public databases the following link was used: https://www.kmplot.com/ 25.

## Results

### CRISPR/Cas9 dropout screening and PSMC6 expression in ovarian carcinoma cell line and specimens

**Figure 1:**
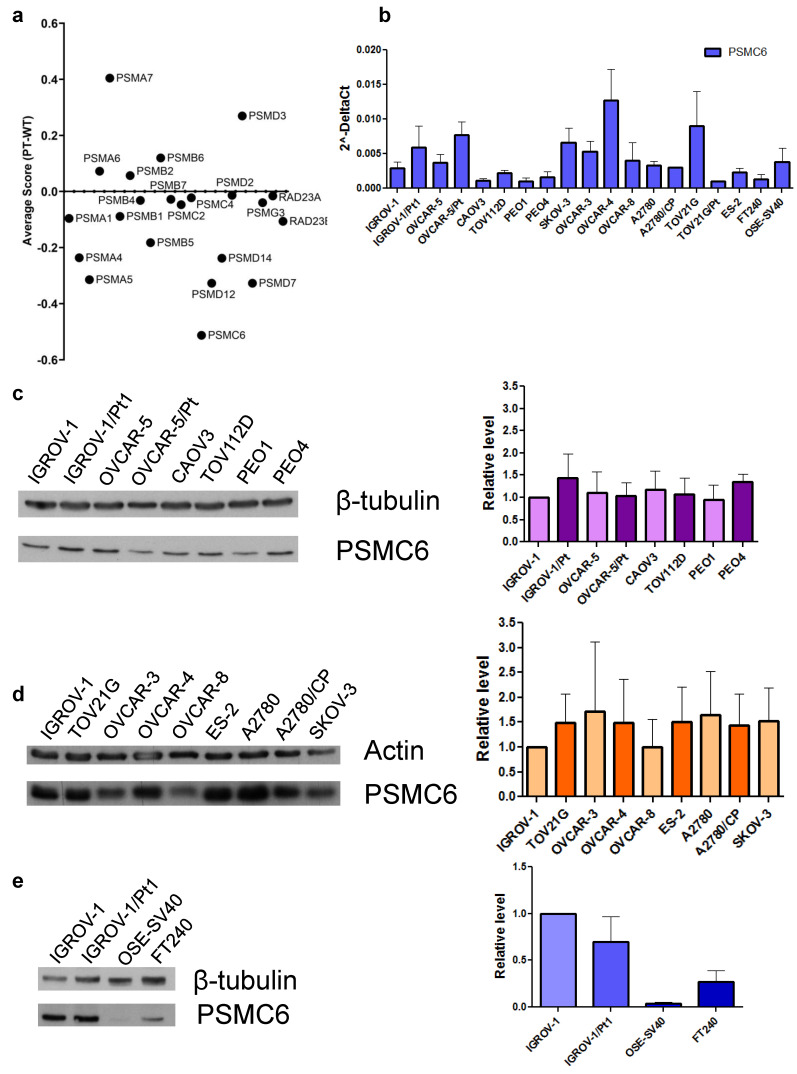
** CRISPR/Cas9 drop out screen and analysis of mRNA and protein levels in a panel of ovarian carcinoma cell lines. a**) Average survival score (ratio) of IGROV-1 (wild-type) cells compared to platinum-resistant (Pt) cells following CRISPR/Cas9 deletion of proteasome subunits. **b**) Levels of PSMC6 mRNAs in a panel of ovarian carcinoma cell lines. Total RNA was extracted from tumor cells and quantified for PSMC6 using qRT-PCR. ΔCt values were obtained by normalizing the Ct values of the target gene to the endogenous control GAPDH. **c**) and **d**) Protein expression of PSMC6 in a panel of ovarian carcinoma cell lines. Protein extracts were analyzed by Western blot. Actin and β-tubulin were used as control for loading. **e**) Protein expression of PSMC6 in IGROV-1, IGROV-1/Pt1, OSE-SV40 and FT240 cells. Protein extracts were analyzed by Western blot. β-tubulin was used as control for loading. The histograms represent the Western blot quantification. The band intensity was quantified by using the ImageQuant software. The intensities of PSMC6 bands were normalized to the intensities of the bands of the corresponding control protein.

The cisplatin-resistant IGROV-1/Pt1 exhibits clinically relevant features such as TP53 mutation that reduces the susceptibility to apoptosis 15 and cell survival pathway activation 17. Therefore, it was selected as a model for the identification of genes key for survival. IGROV-1/Pt1 are 10-fold resistant to cisplatin in comparison to IGROV-1 cells (IC_50_ values by cell growth inhibition assays: 3.4 ± 1.3 vs 0.33± 0.1 µM, respectively) as assessed by cell growth inhibition assays after 72 h drug exposure. CRISPR/Cas9 gene editing allows to pinpoint essential genes for cell survival, exploring distinctions between parental, i.e., sensitive wild-type (Wt) and Pt-resistant cells. Given the importance of the proteasome in maintaining proper protein folding and degradation, we conducted a CRISPR/Cas9 drop-out screen targeting proteasome subunits to assess their impact on ovarian carcinoma cell survival. Following lentiviral transduction with Cas9 and with gRNAs against proteasome components and culture for 21 days, cisplatin-sensitive IGROV-1 and -resistant IGROV-1/Pt1 cells were sequenced to identify genes necessary for survival. The average survival score (ratio) of IGROV-1 cells compared to IGROV-1/Pt1 cells following CRISPR/Cas9 deletion of proteasome subunits is reported in **Figure 1a**. The complete data from the CRISPR/Cas9 dropout screening for both IGROV-1 and IGROV-1/Pt1 cell lines, analyzed independently are reported in **Figure S1**. Notably, our findings reveal that among all tested proteasome subunits, PSMC6 resulted in the most significant lethality score in the IGROV-1/Pt1 cells (average score of -1.20815) compared to parental IGROV-1 cells (average score of -0.694). This result provides a clearer comparison of the effects of PSMC6 loss on cell survival relative to other proteasome subunits. However, the effect observed was more pronounced in resistant cells suggesting a degree of dependency. The ubiquitin receptor Rad23A was also included in the analysis but it was not modulated when comparing sensitive and resistant cells. We therefore explored the expression levels of PSMC6 in a large panel of ovarian carcinoma cell lines representative of different histological subtypes, including A2780, TOV21G, TOV112D, ES-2, OVCAR-3, OVCAR-4, OVCAR-5, OVCAR-8, PEO1, CAOV3, SKOV-3 as well as some resistant variants (A2780/CP, OVCAR-5/Pt, PEO4, TOV21G/Pt) by qRT-PCR and by western blotting. Variable levels of PSMC6 mRNA were found across the cell lines, whereas the protein levels were similar (**Figures 1b and 1c and d**). Markedly reduced PSMC6 protein levels were detected in the immortalized human ovarian (OSE-SV40) and fallopian tube secretory epithelial cells (FT240) (**Figure 1e**).

**Figure 2:**
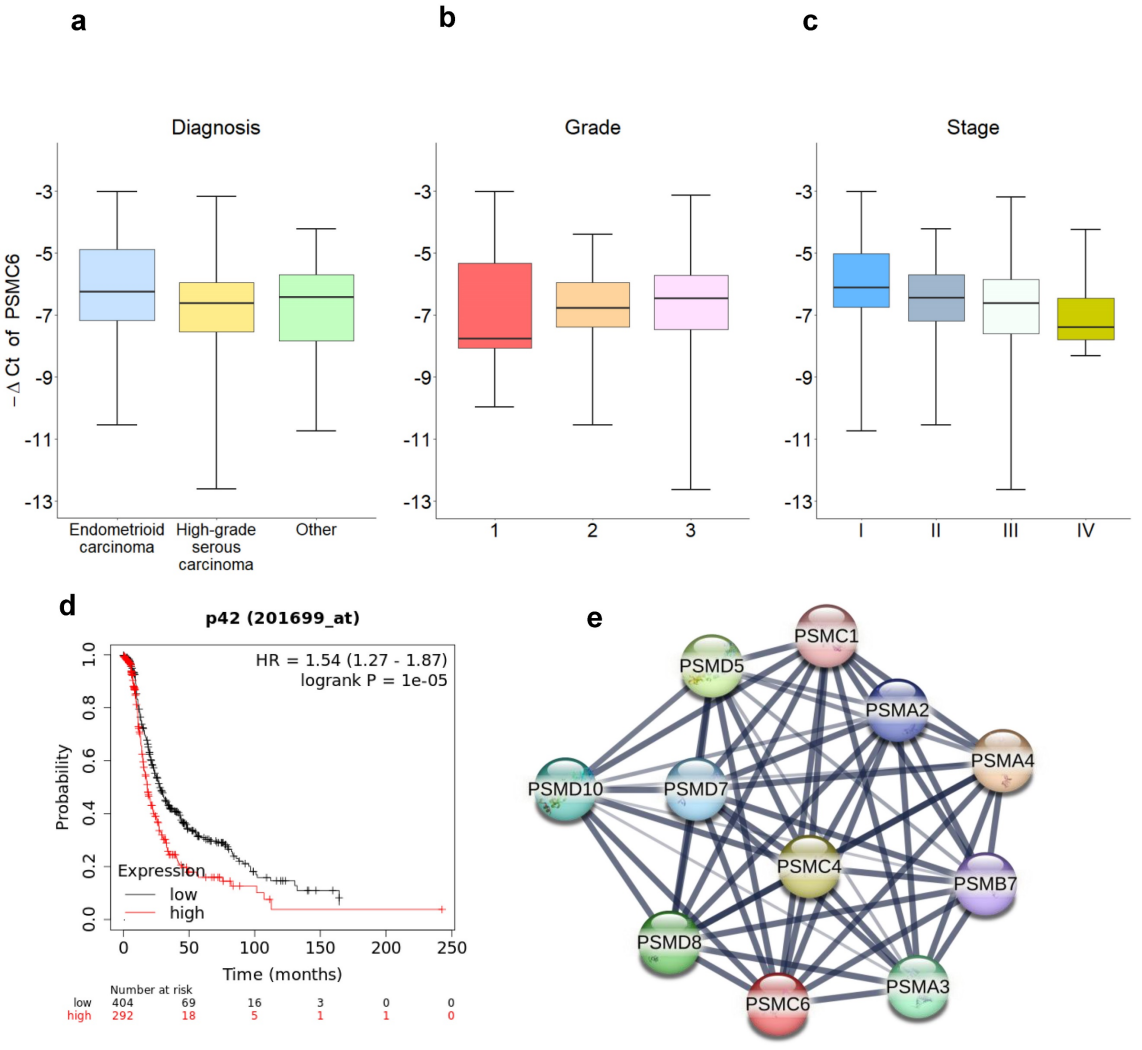
** PSMC6 expression correlations with ovarian cancer. a**), **b**) and** c**) PSMC6 relative expression distribution. Boxplot representing the expression of the PSMC6 (in terms of -ΔCt) according to diagnosis (**a**), tumor grade (**b**) and stage (**c**). Each box indicates the 25th and 75th centiles of the distribution. The horizontal line inside the box indicates the median and the whiskers indicate the extreme measured values. **d**) Progression-free survival of ovarian carcinoma patients from the TCGA case material. **e**) The PSMC6 interaction network built using STRING. The thickness of the lines connecting the hubs represents the edge confidence.

We subsequently evaluated specimens from ovarian carcinoma patients (n= 134) for PSMC6 mRNA levels. Most of the patients (**Table S1**) were high grade serous ovarian carcinoma (60%), Grade 3 (70%) and Stage III (53%). **Table S2** reports the descriptive statistics of the relative expression of PSMC6 according to the diagnosis, tumor grade and stage. No association were found between PSMC6 levels and diagnosis (**Figure 2a**, KW p-value: 0.245) nor grade (**Figure 2b**, KW p-value: 0.408). On the contrary, a borderline significance was found between PSMC6 levels and tumor stage (**Figure 2c**, KW p-value: 0.091). Albeit not statistically significant, this trend was retained also considering the dichotomized stage of disease (I+II vs III+IV) as outcome measure in a univariate logistic regression models (OR: 1.208, 95%CI: 0.944; 1.546).

To further explore the clinical relevance of PSMC6, we interrogated publicly available datasets of ovarian carcinoma. The dataset of the Cancer Genome Atlas (TCGA) Research network 22 was used to investigate PSMC6 expression in relation to clinical follow up in optimally de-bulked patients. In this set, including 696 patients, survival (progression free survival) of patients with high expression of PSMC6 was significantly lower from that of patients with low expression (*P* = 1.0e^-5^, **Figure 2d**).

In addition, we used bioinformatics approaches available from the STRING interaction network (https://string-db.org) to search for functional interactors of PSMC6. The interactors mainly involved other ATPase subunits that like PSMC6 use the energy from ATP hydrolysis to unfold the substrate proteins (**Figure 2e**), suggesting the need of further experimental effort to investigate PSMC6 biological role.

### Functional studies using small interfering RNAs directed to PSMC6

**Figure 3:**
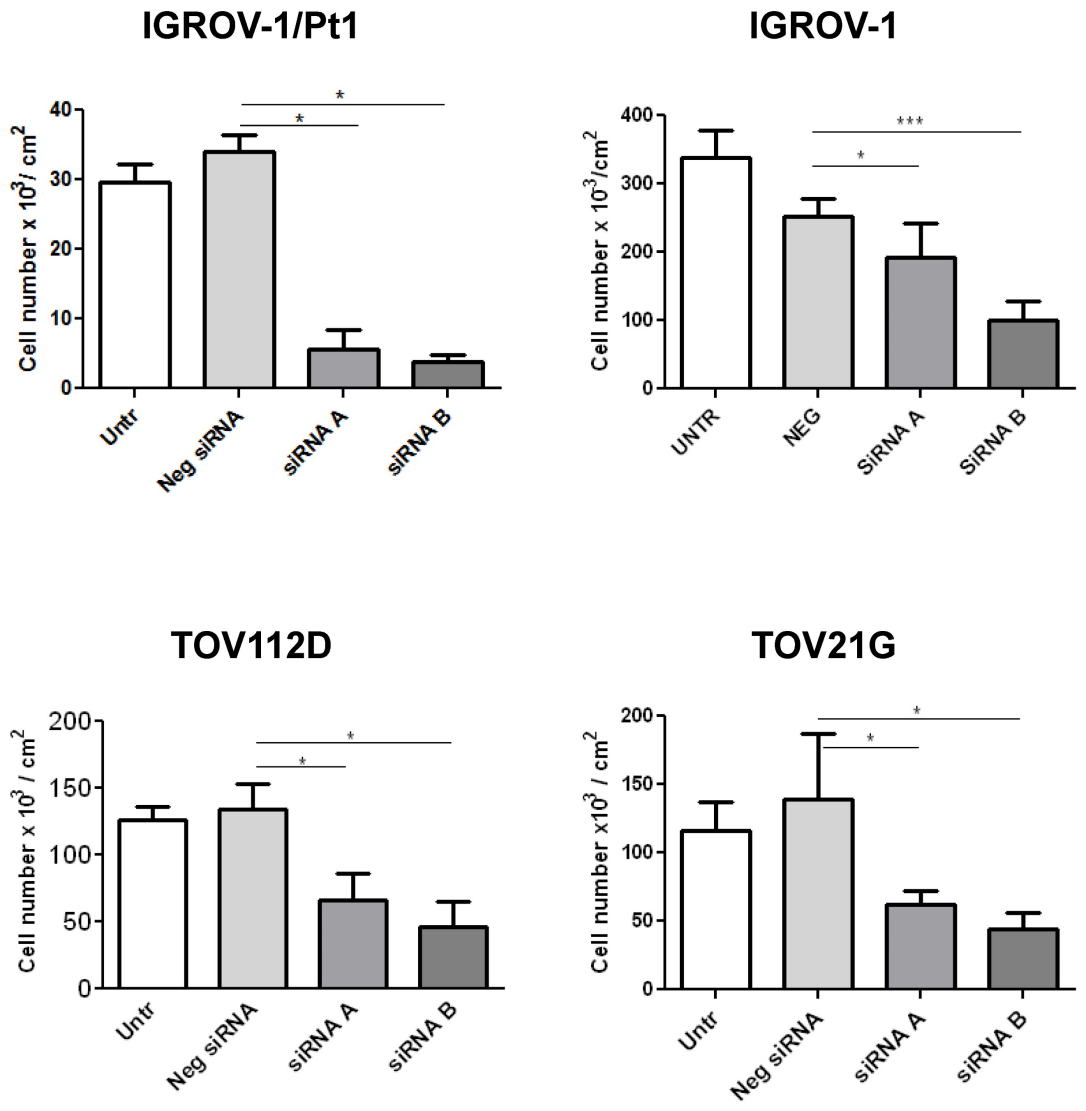
** Effects of PSMC6 silencing on cell growth of IGROV-1, IGROV-1/Pt1, TOV112D and TOV21G.** Measurement of cell density (cells x 10^3^/ cm^2^) after 48 h from transfection in IGROV-1, IGROV-1/Pt1, TOV112D and TOV21G cell lines. Each value is a mean of 3 values from a representative experiment. The statistical analysis was performed by One-way ANOVA test. p < 0.05 were considered significant.

**Figure 4:**
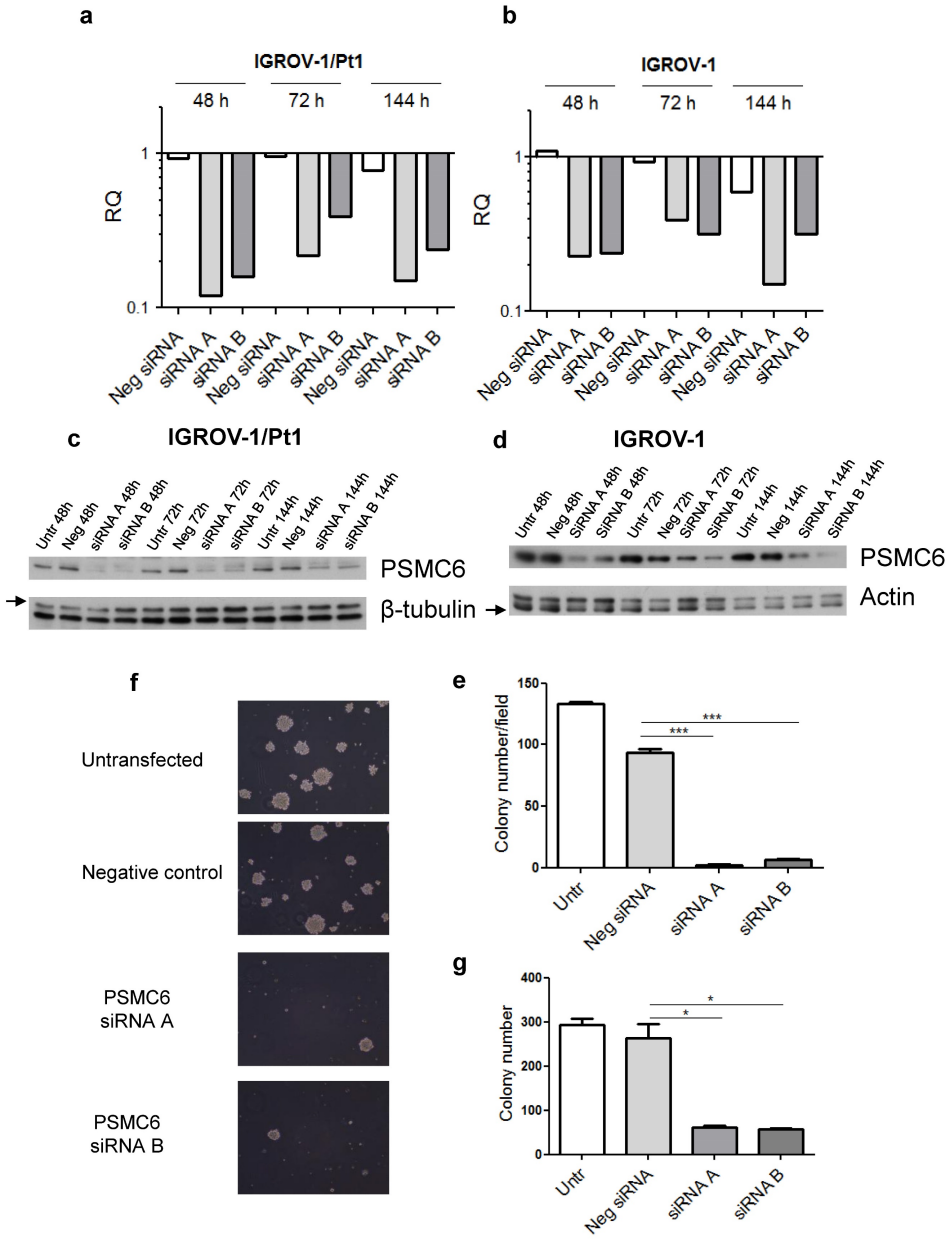
** Efficiency of transient PSMC6 knockdown at mRNA and protein levels in IGROV-1 and IGROV-1/Pt1 cells, and colony forming ability of IGROV-1/Pt1 cells in soft agar and plastic dishes. a**) and **b**) Total RNA was extracted from IGROV-1/Pt1 and IGROV-1 cells and quantified for PSMC6 using qRT-PCR at the experimental times of 48 h, 72 h and 144 h after transfection start using 100 nM of siRNA A and B. ΔCt values were obtained by normalizing the Ct values of the target gene to the endogenous control GAPDH. RQ values are referred to a calibrator, the untransfected cells, whose RQ value was set to 1 at each time point. **c**) and **d**). The expression levels of PSMC6 upon PSMC6 silencing were evaluated using Western blot analysis in IGROV-1/Pt1 and IGROV-1 cells harvested 48 h, 72 h and 144 h after transfection start. The band intensity was quantified for PSMC6 by using the ImageQuant software. Western Blot quantification reported in **Table S3**. **e**) IGROV-1/Pt1 cells were harvested 48 h after siRNA transfection start and they were counted and seeded to evaluate their clonogenic ability in soft agar. The reported values represent the mean ± SD of 6 fields in triplicate from a representative experiment. Kruskal-Wallis test followed by Bonferroni's test (p<0.0001 for Neg siRNA vs siRNA A; p<0.0001 for Neg siRNA vs siRNA B). **f**) Representative images from a colony formation assay carried out in PSMC6-silenced IGROV-1/Pt1 cells. These images were taken 10 days after seeding in soft agar. **g**) Colony forming ability was evaluated in plastic dishes. Cells were harvested 48 h after siRNA transfection start and their colony forming ability was evaluated. The reported values represent the mean ± SD of 3 wells from a representative experiment. Kruskal-Wallis test (p=0.05 for Neg siRNA vs siRNA A; p=0.05 for Neg siRNA vs siRNA B). Untr, untransfected; Neg, negative.

Based on the results from the dropout screens and the above reported findings including investigation of public datasets, we elected to carry out phenotypic analysis. We examined the impact of PSMC6 knockdown in various cell lines by transfecting two siRNAs targeting different regions of the PSMC6 mRNA (**Figure 3**) and the efficiency of PSMC6 silencing has been verified by qRT-PCR (**Figure S2**). We noticed an impact on cell growth in all examined models the highest impact being in IGROV-1/Pt1 cells (**Figure 3**). Then, we focused on the IGROV-1 and IGROV-1/Pt1 pair. PSMC6 mRNA levels were examined 48, 72 and 144 h after start of siRNA A and siRNA B transfection using qRT-PCR. Both siRNAs were found to reduce PSMC6 expression efficiently in IGROV-1/Pt1 and IGROV-1 cell lines, as compared to untransfected cells and cells transfected with a negative control siRNA. The down-regulation of PSMC6 was persistent up to 144 h from transfection start in both cell lines with a slightly more efficient knock-down produced by siRNA A than siRNA B (**Figure 4a, b**).

We next focused on protein expression and examined PSMC6 protein levels in IGROV-1/Pt1 and IGROV-1 cells (**Figure 4c, d**). In IGROV-1/Pt1 cells, the most effective silencing of PSMC6 was observed at 48 and 72 h from transfection start with a percent decrease around 90% (**Table S3**). A less marked down-regulation of PSMC6 protein levels was evidenced at 144 h after transfection start, with still a 60% PSMC6 level decrease (**Figure 4c, Table S3**). Protein levels following transfection with 100 nM siRNA were also evaluated in IGROV-1 cells collected at different time points, i.e., 48, 72 and 144 h (**Figure 4d**). A marked PSMC6 protein down-regulation was evidenced from 48 h, and this behavior persisted up to 144 h after transfection start (**Table S3**).

### Effects of PSMC6 silencing in IGROV-1/Pt1 and additional cells

**Figure 5:**
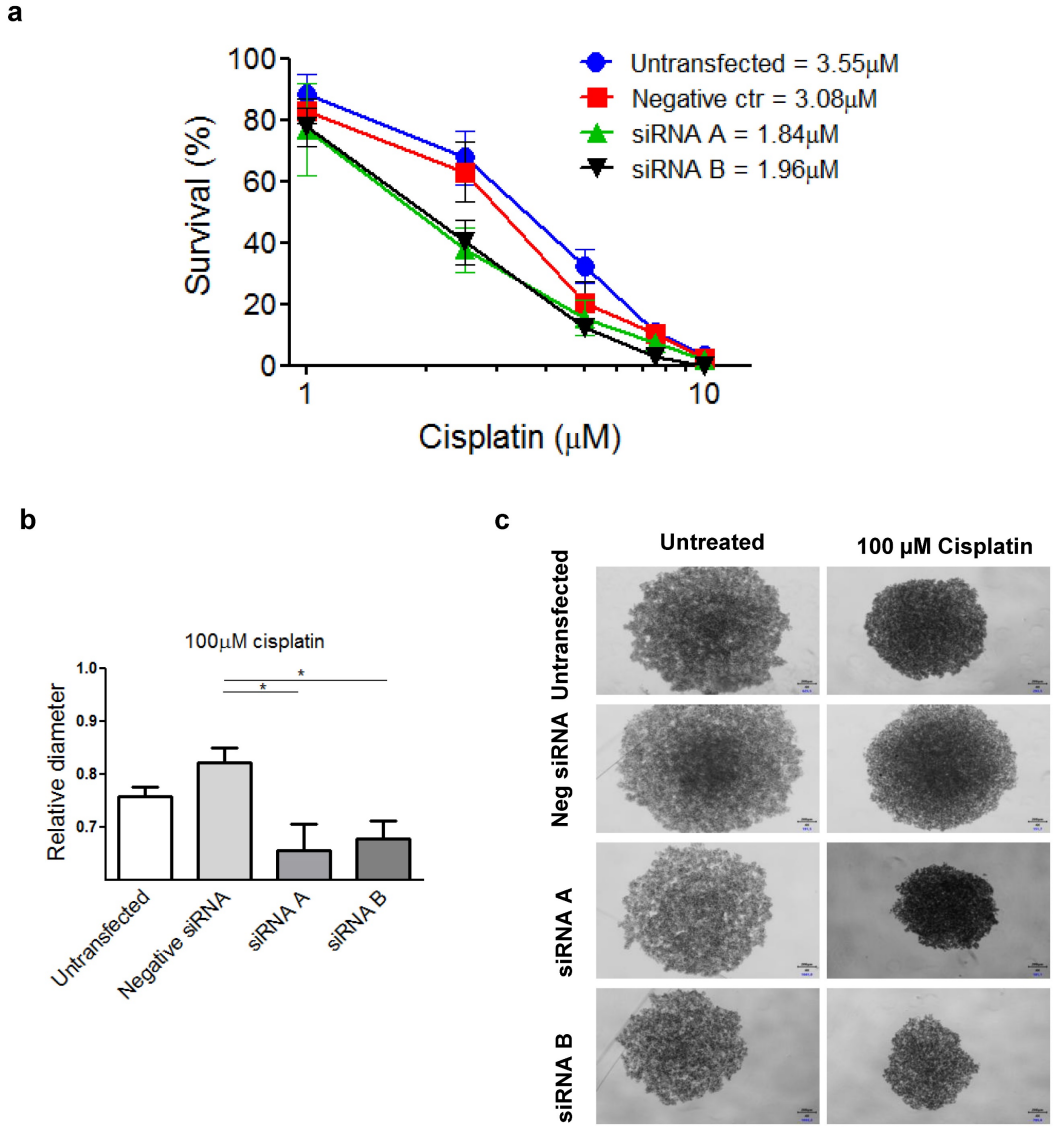
** IGROV-1/Pt1 silencing and its effect on sensitivity to cisplatin by colony forming and spheroid assays. a**) Percentage of survival of IGROV-1/Pt1 cells forming colonies in plastics after treatment with cisplatin at different concentrations. The IC_50_ values for each experimental condition is reported. **b**) Relative diameter of IGROV-1/Pt1 spheroids for the indicated experimental conditions after 72 h treatment with 100 µM cisplatin. The statistical analysis was performed by One-way ANOVA test. p < 0.05 were considered significant. **c**) Representative images of IGROV-1/Pt1 untreated and treated spheroids after 72 h exposure to 100µM cisplatin.

To examine the phenotype of PSMC6-silenced IGROV-1/Pt1 cells, we analysed cell growth and survival. Therefore, 48 h after transfection start, cells were counted to assess the inhibition of proliferation or seeded in soft agar; colonies were microscopically monitored over time and counted using a magnifier 12 days after seeding. An inhibitory effect on clonogenic ability was observed (**Figure 4e, f** and** g**). Compared to negative control transfected cells, a significant decrease of colony forming ability in cells transfected with siRNA A and siRNA B was observed (Kruskal-Wallis test followed by Bonferroni's test). The transfection with siRNA A and B resulted in a 98 and 95 % reduction of the colony number with respect to untransfected cells, while negative control-siRNA transfection produced a slight decrease (29%) with respect to untransfected cells (**Figure 4e**). The impaired clonogenic ability of IGROV-1/Pt1 cells in soft agar is shown in a representative image (**Figure 4f**). A marked effect on clonogenic ability was evidenced upon PSMC6 silencing by colony counting in plastic dishes in both IGROV-1/Pt1 and IGROV-1 cells (**Figure 4g** and **Figure S3a**). As compared to negative control-siRNA transfected cells, a significant decrease of cell number was found in cells transfected with siRNA A and siRNA B (Kruskal-Wallis test). The effect of cisplatin on survival of IGROV-1/Pt1 and IGROV-1 cells was evaluated by colony forming assay. A higher number of cells were seeded for the siRNA A and B transfected compared to untransfected cells due to the PSMC6 knockdown effect. The IGROV-1/Pt1 siRNA-transfected cells were more sensitive to cisplatin than negative control-siRNA transfected and untransfected cells based on the IC_50_ values (**Figure 5a**). This effect was not observed in parental IGROV-1 siRNA-transfected cells. As reported in **Figure S3b**, no change in sensitivity to cisplatin was evidenced in IGROV-1 cells upon PSMC6 silencing. Increased sensitivity to cisplatin in spheroids, evaluated as reduced diameter with respect to the untreated control, was observed in IGROV-1/Pt1 siRNA-transfected cells **(Figure 5b and c)**.

The effect of PSMC6 silencing was evaluated in normal cells (OSE-SV40 and FT240) as well as in other pairs of cisplatin-sensitive and -resistant cell lines (**Figure S4 and S5**). Cell growth inhibition assays were used for normal cells as such cells do not exhibit clonogenic ability. **Figure S4** reports the effect of the silencing of PSMC6 in OSE-SV40 and FT240 cells, no increased sensitivity to cisplatin was recognized upon PSMC6 silencing in comparison to untransfected and negative control transfected OSE-SV40 cells. A slight reduced response to cisplatin was observed in PSMC6-silenced FT240 cells compared to untransfected and negative control transfected cells, consistent with the effect of silencing on cell growth, which is known to impair damage induced by DNA damaging agents. A similar approach applied to OVCAR-5-OVCAR-5/Pt and TOV21G-TOV21G/Pt resulted in no changes in sensitivity to cisplatin following PSMC6 silencing (data not shown). Similar results were obtained using colony forming assay carried out in the same pairs of cell lines except than TOV21G cells, which did not form colonies under our experimental conditions (**Figure S5**).

**Figure 6:**
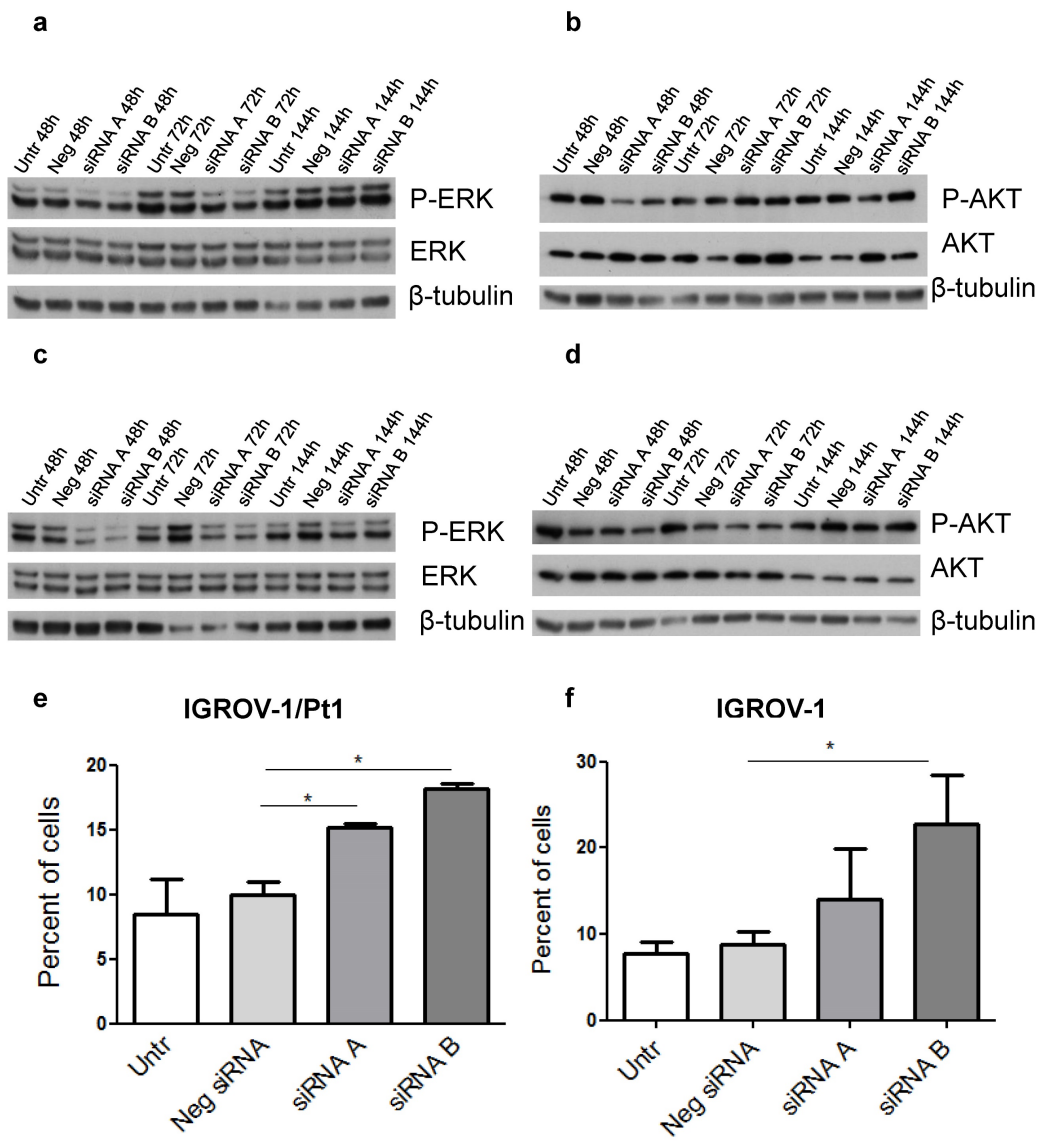
** Western Blot analysis of ERK1/2, AKT and their activation and quantitative analysis of apoptosis in PSMC6-silenced IGROV-1/Pt1 and IGROV-1 cells.** IGROV-1/Pt1 (**a**,**b**) and IGROV-1 (**c**,**d**) cells were harvested 48 h, 72 h and 144 h after transfection start for protein analysis. Cell lysates were analyzed by Western blotting. The band intensity was quantified for P-ERK1/2, ERK1/2, P-AKT and AKT by using the software ImageQuant. Western Blot quantification reported in **Table S4**. **e**), **f**) For apoptosis, cells were silenced and 72 h after transfection of siRNAs, they were harvested for the Annexin V-binding assay. The shown values are the mean ± SD from 3 replicates of a representative experiment. Kruskal-Wallis test was applied to compare all samples (IGROV-1/Pt1: p=0.05 for Neg siRNA vs siRNA A; p=0.05 for Neg siRNA vs siRNA B; IGROV-1: p=0.1 for Neg siRNA vs siRNA A; p=0.05 for Neg siRNA vs siRNA B).

Because a major impact on tumour cell growth and survival was evidenced by the initial phenotypic analysis, we elected to focus on the two major cellular hubs i.e., the MAPK and PI3K/AKT pathways. In PSMC6-silenced IGROV-1/Pt1 cells, a marked down-regulation of the canonical MAPK pathway, supported by the decreased levels of phospho-ERK1/2 (P-ERK1/2^Thr202/Tyr204^) was evidenced with a 41 and 68% decrease at 48 and 72 h after transfection start (**Figure 6a**, **Table S4**). Conversely, a slight decrease of phosphorylated Akt (P-AKT^ Ser473^) and AKT levels was detected following 48 h transfection (**Table S4**). Such a modulation was not maintained at 72 h, thereby implying a transient effect (**Table S4, Figure 6b**). Regarding IGROV-1 cells, a reduced phosphorylation of ERK1/2^Thr202/Tyr204^ was observed at different time points, supporting a suppression of the MAPK pathway (**Table S4**, **Figure 6c**). Instead, only a modest decrease of the P-AKT^Ser473^ levels was evidenced with siRNA B (**Figure 6d**). Indeed, the negative control-siRNA transfected cells already displayed decreased phosphorylation of P-AKT^Ser473^. No changes in AKT levels were observed. We carried out a validation of the phosphorylation of ERK1/2 in IGROV-1 cells by ELISA and we confirmed a down modulation of P-ERK1/2 activation (**Figure S6**).

To further investigate the phenotypic features of PSMC6-silenced IGROV-1/Pt1 and IGROV-1 cells, apoptosis levels were quantitatively measured using the Annexin V binding assays. A slight increase of apoptotic cells in PSMC6-silenced IGROV-1/Pt1 and IGROV-1 cells was evidenced using both siRNAs 72 h after siRNA transfection (**Figure 6e-f**).

### Analysis of the expression of DUSP5, DUSP6 and ubiquitinated protein accumulation levels in PSMC6-silenced IGROV-1 and IGROV-1/Pt1 cells

**Figure 7:**
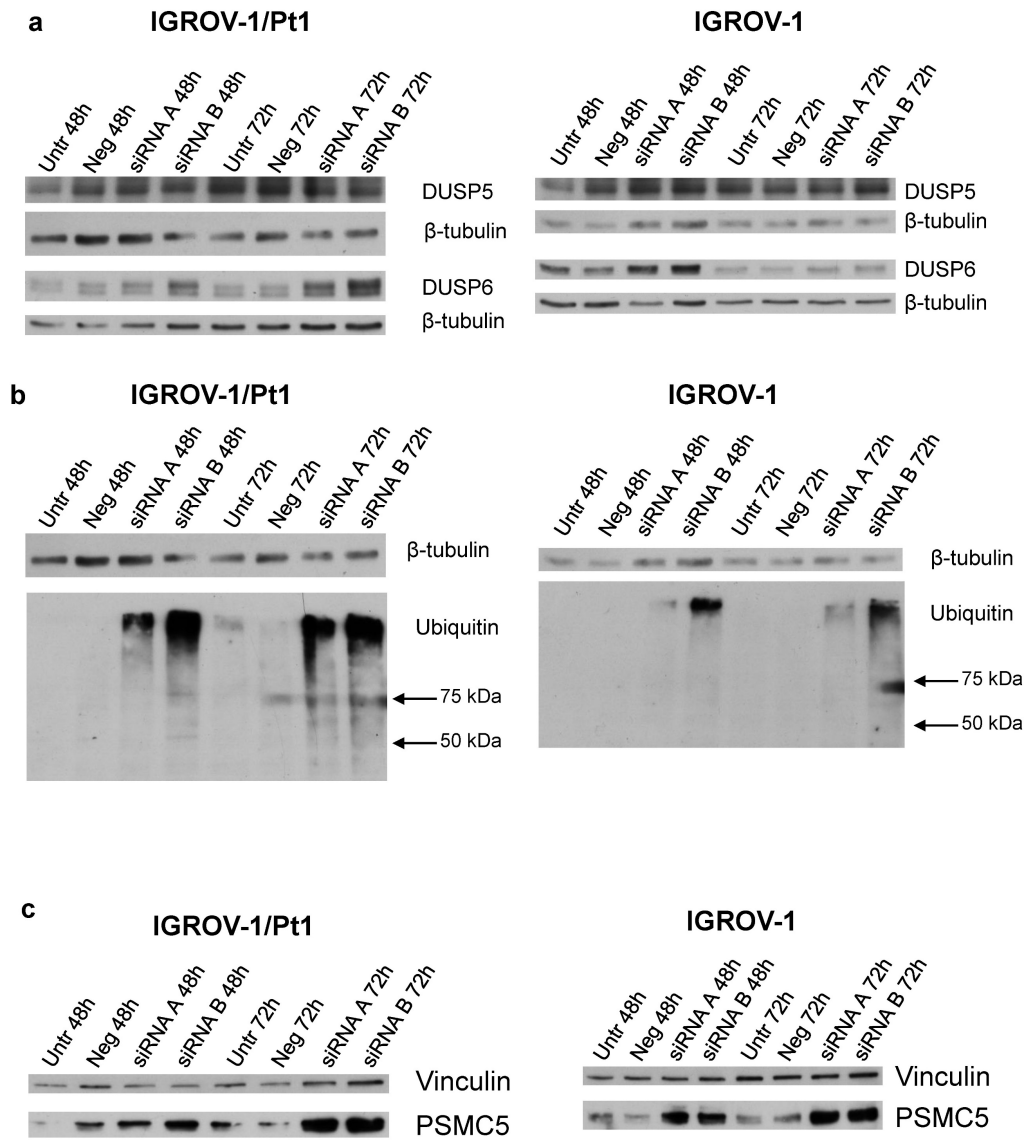
** Western Blot analysis of DUSP5, DUSP6, ubiquitinated protein levels and PSMC5 proteasome subunits in PSMC6-silenced IGROV-1/Pt1 and IGROV-1 cells.** IGROV-1/Pt1 and IGROV-1 cells were harvested 48 h and 72 h after transfection start for protein analysis. **a**) Cell lysates were analyzed by Western blotting for DUSP5 and DUSP6. **b**) IGROV-1/Pt1 and IGROV-1 cells were harvested 48 h and 72 h after transfection start for ubiquitinated protein accumulation analysis. Cell lysates were analyzed by Western blotting. β-tubulin represents control for loading. **c)** Western blot analysis of PSMC5 in IGROV-1/Pt1 and IGROV-1 cells. The band intensity was quantified by using the ImageQuant software. Western Blot quantifications are reported in **Table S5**.

To clarify the mechanisms of MAPK deregulation observed upon PSMC6 silencing in IGROV-1/Pt1 and IGROV-1 cells, the relevance of ERK1/2 phosphatases was explored. Indeed, the phosphatase activities of DUSP5 and DUSP6 are reported to specifically deactivate ERK1/2 26. Western blot analysis of DUSP5 levels in PSMC6-silenced cells indicated a modest modulation not really supporting a critical role in controlling P-ERK1/2^Thr202/Tyr204^ levels in these models (**Figure 7a** and **Table S5**). On the contrary, the level of DUSP6 was increased upon silencing, implying that the observed down-regulation of P-ERK1/2^Thr202/Tyr204^ levels could be explained by the phosphatase accumulation (**Figure 7a** and **Table S5**).

To assess whether PSMC6 knockdown resulted in an impairment of the ubiquitination process, an anti-ubiquitin antibody in both PSMC6-silenced sensitive and resistant cell models was used (**Figure 7b**). The knockdown of PSMC6 resulted in an accumulation of ubiquitinated proteins in PSMC6-silenced cell systems at 48 and 72 h after transfection start, suggesting that PSMC6 is required for a correct protein degradation to occur.

We investigated whether the PSMC6 knockdown was associated to a concomitant variation of other PSMC proteins. Overall, PSMC6 silencing was not associated with down-regulation or up-regulation of other PSMC proteins (data not shown) except than PSMC5 in IGROV-1/Pt1 cells 72 h after transfection start (**Figure 7c** and **Table S5**).

### Analysis of apoptosis-related proteins by antibody arrays

To further explore the phenotypic changes occuring upon PSMC6 knockdown in ovarian carcinoma cells, we used an antibody array approach in PSMC6-silenced IGROV-1/Pt1 and IGROV-1 cells to identify perturbations of factors implicated in cell growth and apoptosis induction. As shown by the heatmaps (**Figure S7a** and** b, Figure S8**) several modulated proteins were detected upon knockdown. In the cisplatin-resistant cells, PSMC6 silencing resulted in increased expression of p21^cip1/waf1^, p27^kip1^, and cleaved Caspase-3. Alterations of the levels of BAX and BCL2 were also evidenced upon PSMC6 silencing. In IGROV-1 cells, the most striking changes were the up-modulation of p21^cip1/waf1^ and of cleaved caspase 3. To validate the results observed by protein arrays, Western blot analysis was performed. An upmodulation of p21^cip1/waf1^ and cleaved caspase-3 was evident in IGROV-1/Pt1 cells (**Figure S7c**, **Table S6**). In IGROV-1 cells, the pattern of modulation of p21^cip1/waf1^ reflected what observed with the protein arrays (**Table S6**).

### PSMC6 interaction network

Following the biochemical analysis carried out in this study, the interaction network of PSMC6 (**Figure 2e**) could be enriched with CDKN1A and B, DUSP5 and 6, MAPK1 and 3, and CASP3 (**Figure S9**).

### Cell sensivity to proteasome inhibitors

To explore the pharmacological potential of PSMC6 targeting, we tested the efficacy of drugs directed to the proteasome following PSMC6 knockdown in IGROV-1/Pt1 and IGROV-1 cells using growth inhibition assays. To this end, cells were seeded at higher density to cope with the growth inhibitory effect of PSMC6 knockdown. Under our experimental conditions no change in sensitivity was observed both when using bortezomib, which targets the catalytic function of the proteasome residing in the 20S core particle, and bAP15, which affects proteasome by interference with the 19S regulatory particle (**Figure S10**). Such results suggest that PSMC6 targeting represent a new mechanism for blocking proteasome function.

## Discussion

There is an urgent need to define alterations conferring survival to ovarian cancer cells to explore possible therapeutic targeting. The CRISPR/Cas9 gene editing allows to identify genes critical in cell survival. Given the relevance of the proteasome in maintaining correct protein folding, a feature altered in cancer cells, we performed a CRISPR/Cas9 drop out screen for proteasome subunits to examine their contribution to ovarian carcinoma cell survival. Cisplatin-sensitive IGROV-1 and -resistant IGROV-1/Pt1 ovarian carcinoma cell lines were used because the resistant variant displays clinically relevant features, i.e. *TP53* mutation, survival pathway deregulation 15, 17, 27, 28. Since the drop out screen suggested a role for PSMC6 in survival of IGROV-1/Pt1 cells, we examined publicly available data set and found that PSMC6 expression was associated with reduced progression free survival of ovarian cancer patients, supporting the interest of further exploring its biological significance. PSMC6 was found expressed in several cancer cell lines with enhanced levels as compared to normal ovarian and fallopian tube cells. PSMC6 loss negatively impacted on cell growth and clonogenic ability and favoured apoptosis in IGROV-1/Pt1 cells. A significant increase in cisplatin sensitivity was observed upon PSMC6 silencing both in clonogenic and spheroid assays. A marked down regulation of the canonical MAPK pathway was observed, as supported by decreased phospho-ERK1/2 (P-ERK1/2^Thr202/Tyr204^) in PSMC6-silenced IGROV-1/Pt1 cells. The phenotypes of resistant cells were also observed in the parental cells with a less marked impact of PSMC6 silencing on cell growth and a lack of change in sensitivity to cisplatin, implying less dependence of parental cells on the canonical MAPK pathway for survival. The effect of PSMC6 silencing on the canonical MAPK pathway suggested a stabilization of the phosphatases that regulate ERK1/2 activation, with DUSP6 enhancement. Given that PSMC6 acts in concert with other proteasome regulatory particle subunits to allow the gate opening to translocate unfolded substrates to the proteolytic proteasome chamber, the down-regulation of its expression reasonably results in impaired protein transfer, with accumulation of various ubiquitinated proteins. Such an accumulation was indeed observed upon PSMC6 silencing in cisplatin-sensitive and -resistant cells. We think that following ubiquitination, several proteins are accumulated and it is likely that the accumulation of ubiquitinated DUSP6 may explain phospho-ERK decrease.

An analysis of the impact of PSMC6 knockdown in additional drug-resistant ovarian carcinoma cell lines indicated a lack of sensitization to cisplatin in models other than IGROV-1/Pt1. Since IGROV-1/Pt1 cells are of endometroid nature, our findings support a certain dependence of sensitization upon PSMC6 knockdown on the histotype and its molecular features. In this regard, a key point is the link between cisplatin resistance and MAPK pathway activation in IGROV-1/Pt1 cells 17 as well as the relevance of pathway alterations in endometroid tumors 29. This hypothesis is corroborated by the evidence that, on the contrary to what observed in IGROV-1/Pt1, in serous ovarian carcinoma OVCAR-5/Pt cells the silencing of PSMC6 produced an increased activation of the MAPK pathway that likely favours cell survival counteracting cisplatin cytotoxicity (**Figure S11**).

Of note, PSMC6 silencing did not increase sensitivity to cisplatin in normal cells, where the expression of the gene is quite reduced as compared to tumor cells. Such a feature indicates a promising window of selectively for future PSMC6-targeting approaches.

An additional feature of PSMC6 knockdown cells was an increased level of apoptosis as shown by Annexin V- binding assays, particularly evident in IGROV-1/Pt1 cells. The impact of PSMC6 on apoptosis may vary in different scenarios. The knockdown of PSMC6 impacted on the phenotype of IGROV-1 cells as well. The result of the CRISPR/Cas9 screen is the ratio between genes of IGROV-1 and IGROV-1/Pt1 cells. Although PSMC6 is important for both the cell lines, the effect on viability was much more pronounced in IGROV-1/Pt1 cells indicating a degree of dependency. Overall, this finding suggests a key physiological role for PSMC6 in multiple biological functions. Indeed, embryonic lethality prior to organogenesis occurs in homozygous knockout mice (mousephenotype.org), supporting an essential role for PSMC6 not consistently rescued by compensatory increase of other 19S regulatory particle subunits. Consistently, silencing of PSMC6 resulted in a mild up-regulation of only one of the ATPase subunits, i.e., PSMC5 at a single time point in IGROV-1/Pt1 cells.

**Figure 8:**
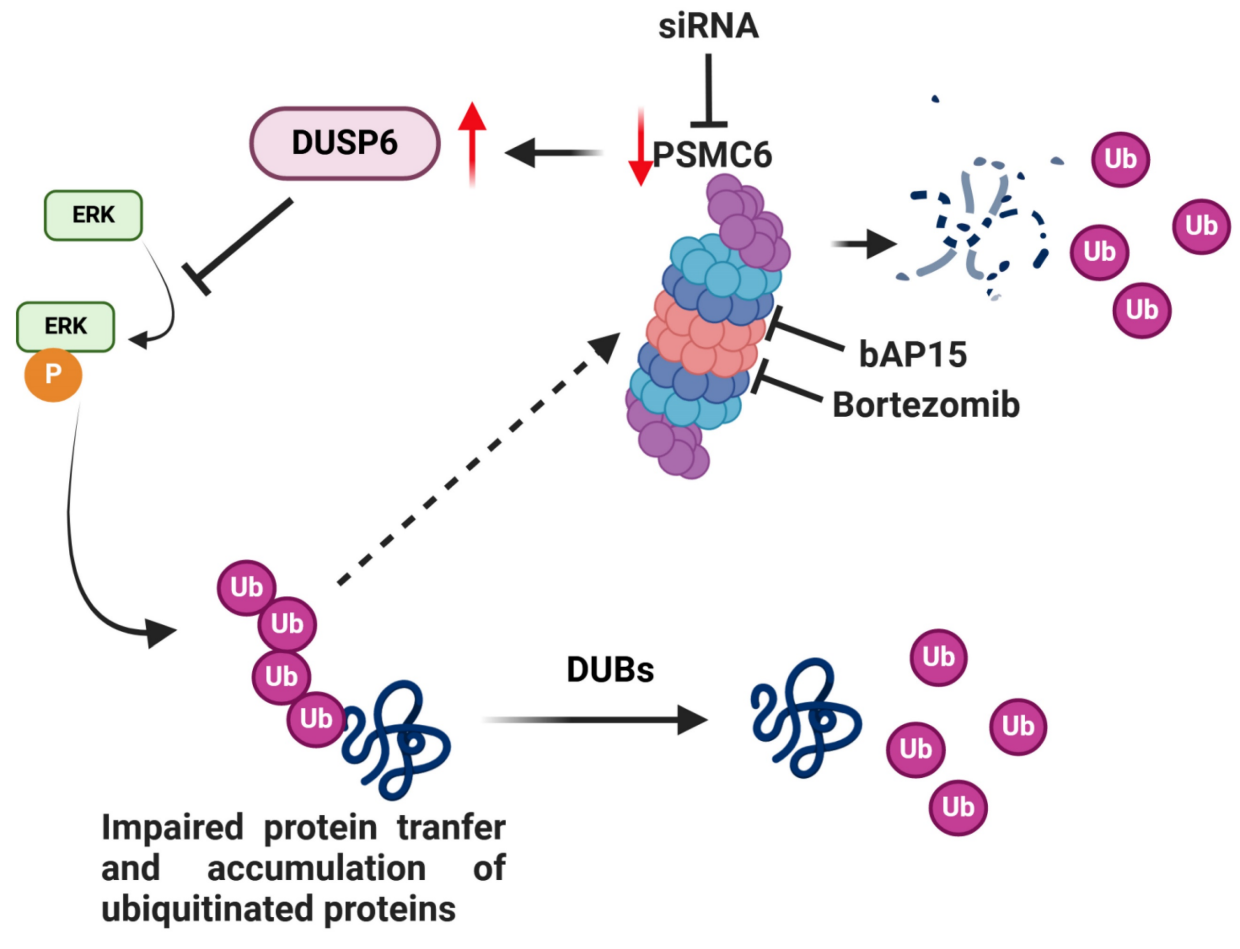
** Model for the effects of targeting of PSMC6 in ovarian carcinoma.** The silencing of PSMC6 is associated with marked down regulation of the activation of the canonical MAPK pathway and an increase of the specific ERK1/2 DUSP6 phosphatase. PSMC6 knockdown appears to lead to impaired protein trasfer to proeasome and accumulation of ubiquitinated proteins. The figure also reports the two proteasome inhibitors bAP15 and bortezomib. Created with BioRender.com.

A comparison of the response to PSMC6 knockdown by antibody arrays and validation by western blotting provided evidence that sensitive and resistant cells display a different response with a preferential activation of apoptosis in IGROV-1/Pt1 resistant cells and of cell cycle arrest in parental cells as shown by caspase 3 cleavage and up-regulation of p21. An analysis of the interactive network of PSMC6 by bioinformatic tools indicated that other ATPase subunits of the 19S regulatory proteasome particle interact with PSMC6. Such evidence together with our results showing that PSMC6 knockdown cells do not modulate their sensitivity to the inhibitor of the enzymatic proteasome activity bortezomib nor to the inhibitor of the 19S associated deubiquitinase USP14 bAP15, support the view that a third mode of proteasome inhibition can be defined by our study. Indeed, interference with an ATPase subunit can represent an additional strategy to hit the protein degrading apparatus of tumor cells. An integrated model of proteasome targeting approaches is provided in **Figure 8**. Although the essential physiological function of PSMC6 protein may discourage from investigating PSMC6 druggability, the increased expression observed in our tumor model systems as compared to normal cells supports the interest of PSMC6 targeting. We also carried out an analysis of PSMC6 expression in ovarian carcinoma clinical specimens in which we observed that earlier stage disease tended to display higher PSMC6 levels, suggesting to explore approaches for targeting PSMC6 in early-stage tumors.

Thus, PSMC6 contributes to maintain the growth/survival of ovarian carcinoma cells. Little information is available on PSMC6 in cancer. PSMC6 contributes to poor overall survival of lung cancer patients 30, 31. In lung adenocarcinoma, PSMC6 was found upregulated as compared to normal adjacent tissues 31, 32 - an observation that reflects our results on levels in normal ovarian and tumor cells - and PSMC6 silencing reduced the growth, migratory and invasive abilities of lung cancer cells 32. PSMC6 has been shown to promote osteoblast apoptosis by inhibiting the PI3K-Akt pathway 33, supporting the relevance of the biological context in determining the protein role.

## Conclusions

In conclusion, the results obtained in this study support that PSMC6 plays a major role in the survival of ovarian carcinoma cells particularly in cisplatin-resistant cells of the endometrioid histotype, through a modulation of the MAPK pathways. Such results suggest the interest of proteasome as a target for antitumor agents in keeping with the available literature, but add a new opportunity for therapeutic targeting. Here, a new mode of proteasome targeting different from the inhibition of the 20S core catalytic activity and from the 19S-associated deubiquitinase activity is suggested, supporting that a better understanding of the key factors involved in maintaining the survival of drug-resistant cells may be helpful in the design of innovative therapeutic strategies.

## Supplementary Material

Supplementary figures and tables.

## Abbreviations

20S CP: 20S proteasome core particle
FBS: fetal bovine serum
KW: Kruskal-Wallis
PARP: Poly-ADP-ribose polymerase
PSMC6: Proteasome 26S Subunit, ATPase 6
Pt: platinum
qRT-PCR: quantitative Real Time PCR
Rpt1-6: Regulatory Particle Triple-A ATPases
siRNAs: small interfering RNAs
USP: Ubiquitin Proteasome System
W: non-parametric Wilcoxon
