# Supplementary material for: PSMC6 regulation of ovarian cancer cisplatin resistance unravels a new mode for proteasome targeting
Source: Int J Biol Sci. 2025 Feb 26;21(5):2258–74. doi: 10.7150/ijbs.104612 (PMC11900818; doi:10.7150/ijbs.104612)

Supplementary materials

PSMC6 regulation of ovarian cancer cisplatin resistance unravels a new mode for proteasome targeting

Matteo Costantino[a‡], Luca Mirra[a‡], Padraig D'arcy[b], Cristina Corno[a], Nives Carenini[a], Elisabetta Corna[a], Johannes Gubat[b], Chiara M. Ciniselli[c], Pietro Pratesi[c], Paolo Verderio[c], Stig Linder[b,d], Giovanni L. Beretta[a*], Paola Perego[a]

[a]Unit of Molecular Pharmacology, Department of Experimental Oncology, Fondazione IRCCS Istituto Nazionale dei Tumori, Milan, Italy. matteo.costantino@istitutotumori.mi.it; luca.mirra@istitutotumori.mi.it; cristina.corno@istitutotumori.mi.it; nives.carenini@istitutotumori.mi.it; elisabetta.corna@istitutotumori.mi.it; paola.perego@istitutotumori.mi.it

[b]Department of Biomedical and Clinical Sciences, Linköping University, Sweden. padraig.darcy@liu.se; johannes.gubat@liu.se

[c]Unit of Bioinformatics and Biostatistics, Department of Epidemiology and Data Science, Fondazione IRCCS Istituto Nazionale dei Tumori, Via Venezian, 1, 20133 Milan, Italy. chiara.ciniselli@istitutotumori.mi.it; pietro.pratesi@istitutotumori.mi.it; paolo.verderio@istitutotumori.mi.it

[d]Division of Biochemistry, Department of Medical Biochemistry and Biophysics, Karolinska Institutet, Stockholm, Sweden. stig.linder@liu.se

[‡]Co-first authors

[*]Corresponding author at: Fondazione IRCCS Istituto Nazionale dei Tumori, Via Amadeo 42, 20133 Milan, Italy. Phone:+39.02.23903080; email address: giovanni.beretta@istitutotumori.mi.it

Table S1. Characteristics of patients involved in the pilot study

	N	%
Diagnosis		
Endometrioid carcinoma	25	18.66
High-grade serous carcinoma	80	59.70
Other[a]	29	21.64
Grade		
1	15	11.19
2	13	9.70
3	106	79.10
Stage		
I	26	19.40
II	21	15.67
III	71	52.99
IV	16	11.94

[a] Other, Mucinous adenocarcinoma, Clear cell carcinoma, Low-grade serous carcinoma

Table S2. Descriptive statistics of the relative expression of PSMC6 according to the diagnosis, tumor grade and stage.

	N	Min	Q1	Median	Q3	max	IQR
**Diagnosis**							
Endometrioid carcinoma	25	-10.55	-7.19	-6.25	-4.88	-3.02	2.31
High-grade serous carcinoma	79	-12.62	-7.57	-6.61	-5.95	-3.19	1.62
Other	29	-10.74	-7.85	-6.43	-5.71	-4.22	2.14
**Grade**							
1	15	-9.96	-8.11	-7.75	-5.31	-3.02	2.80
2	13	-10.55	-7.38	-6.76	-5.96	-4.41	1.43
3	105	-12.62	-7.46	-6.46	-5.72	-3.14	1.74
**Stage**							
I	26	-10.74	-6.76	-6.1	-4.92	-3.02	1.84
II	21	-10.55	-7.19	-6.43	-5.71	-4.22	1.48
III	71	-12.62	-7.63	-6.61	-5.81	-3.19	1.82
IV	15	-8.31	-7.85	-7.38	-6.42	-4.25	1.43

Table S3. Western blot quantification of Figure 4 as percentage of band intensity relative to negative control band at each time point[a].

Table S1						
**PSMC6**	**siRNA A 48h**	**siRNA B 48h**	**siRNA A 72h**	**siRNA B 72h**	**siRNA A 144h**	**siRNA B 144h**
**IGROV-1/Pt1**	8	7	9	12	33	36
**IGROV-1**	21	38	34	21	27	7

[a] The band intensity was quantified by using ImageQuant software, intensities were normalized to the control protein β-tubulin or actin; the siRNAs percentage of intensity was calculated by dividing their normalized band intensity to their negative control normalized band intensity of the same time point.

Table S4. Western blot quantification of Figure 6 as percentage of band intensity relative to negative control band at each time point[a].

Table S2						
**AKT**	**siRNA A 48h**	**siRNA B 48h**	**siRNA A 72h**	**siRNA B 72h**	**siRNA A 144h**	**siRNA B 144h**
**IGROV-1/Pt1**	165	211	210	211	210	128
**IGROV-1**	106	110	82	99	166	185
**P-AKT**	**siRNA A 48h**	**siRNA B 48h**	**siRNA A 72h**	**siRNA B 72h**	**siRNA A 144h**	**siRNA B 144h**
**IGROV-1/Pt1**	45	113	121	112	70	100
**IGROV-1**	119	86	71	98	120	173
**ERK1/2**	**siRNA A 48h**	**siRNA B 48h**	**siRNA A 72h**	**siRNA B 72h**	**siRNA A 144h**	**siRNA B 144h**
**IGROV-1/Pt1**	110	90	82	73	83	67
**IGROV-1**	106	114	97	100	155	164
**P-ERK1/2**	**siRNA A 48h**	**siRNA B 48h**	**siRNA A 72h**	**siRNA B 72h**	**siRNA A 144h**	**siRNA B 144h**
**IGROV-1/Pt1**	80	60	60	50	100	75
**IGROV-1**	42	19	22	29	36	154

[a] Quantification procedure as stated in Table S3.

Table S5. Western blot quantification of Figure 7 as percentage of band intensity relative to negative control band at each time point.

Table S3				
**DUSP5**	**siRNA A 48h**	**siRNA B 48h**	**siRNA A 72h**	**siRNA B 72h**
**IGROV-1/Pt1**	122	244	157	100
**IGROV-1**	47	33	71	121
**DUSP6**	**siRNA A 48h**	**siRNA B 48h**	**siRNA A 72h**	**siRNA B 72h**
**IGROV-1/Pt1**	94	112	220	300
**IGROV-1**	357	243	171	200
**PSMC5**	**siRNA A 48h**	**siRNA B 48h**	**siRNA A 72h**	**siRNA B 72h**
**IGROV-1/Pt1**	228	370	310	228
**IGROV-1**	502	413	304	259

[a] Quantification procedure as stated in Table S3.

Table S6. Western blot quantification of Figure S7c as percentage of band intensity relative to negative control band.

Table S4		
**p21**	**siRNA A**	**siRNA B**
**IGROV-1/Pt1**	250	133
**IGROV-1**	114	214
**Cleaved caspase-3**	**siRNA A**	**siRNA B**
**IGROV-1/Pt1**	143	221
**IGROV-1**	217	192

[a] Quantification procedure as stated in Table S3.

Figure S1: Complete data from the CRISPR/Cas9 dropout screening for both IGROV-1 and IGROV-1/Pt1 cell lines, analyzed independently. Notably, our findings reveal that among all tested proteasome subunits, PSMC6 resulted in the most significant lethality score in the IGROV-1/ Pt cells (average score of -1.20815) compared to IGROV-1 cells (average score of -0.694).

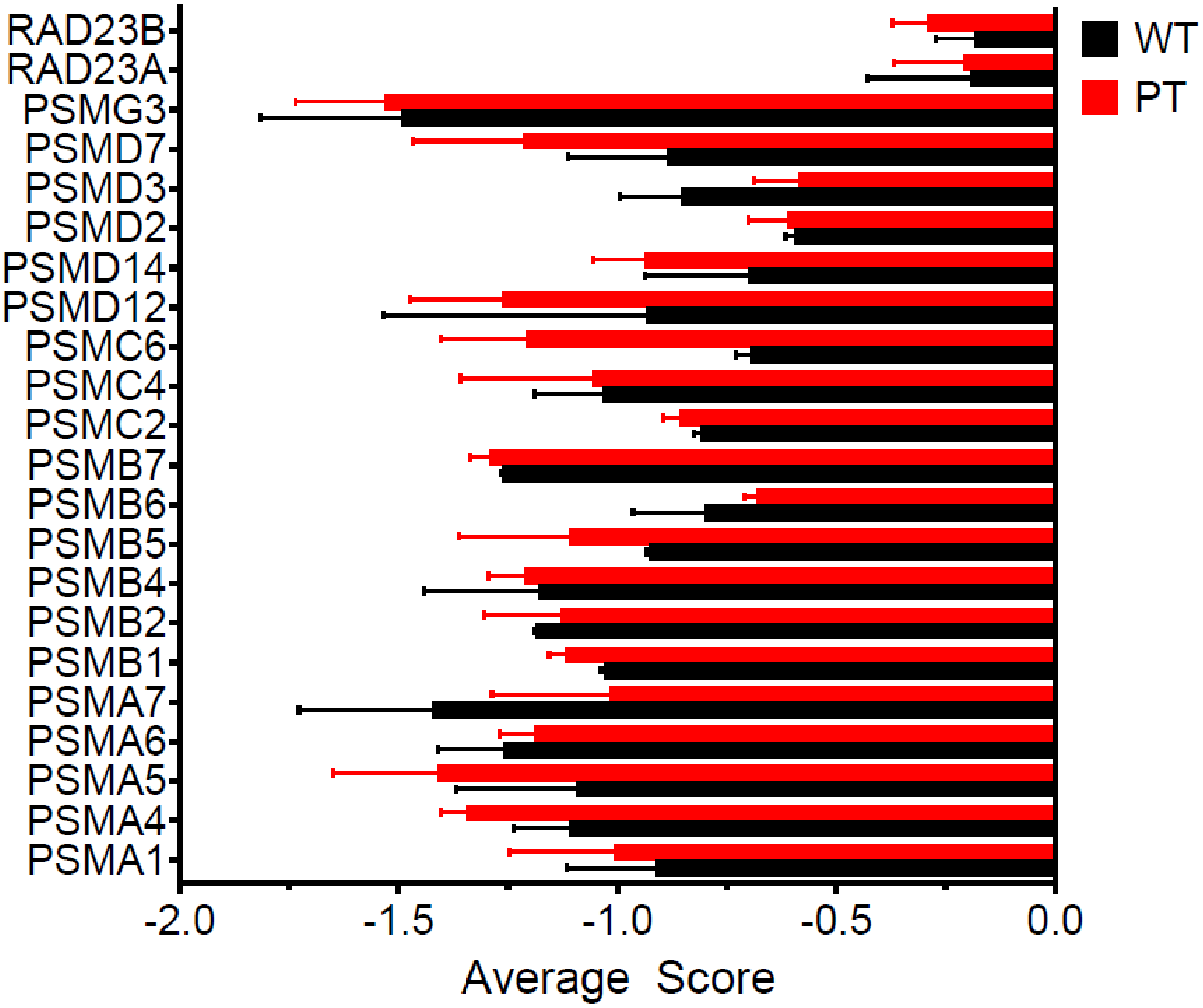

Figure S2: qRT-PCR of PSMC6 in silenced ovarian cancer cell lines. RNA of siRNA transfected cells was extracted and retrotranscribed into cDNA for RT-PCR for PSMC6 and GAPDH.

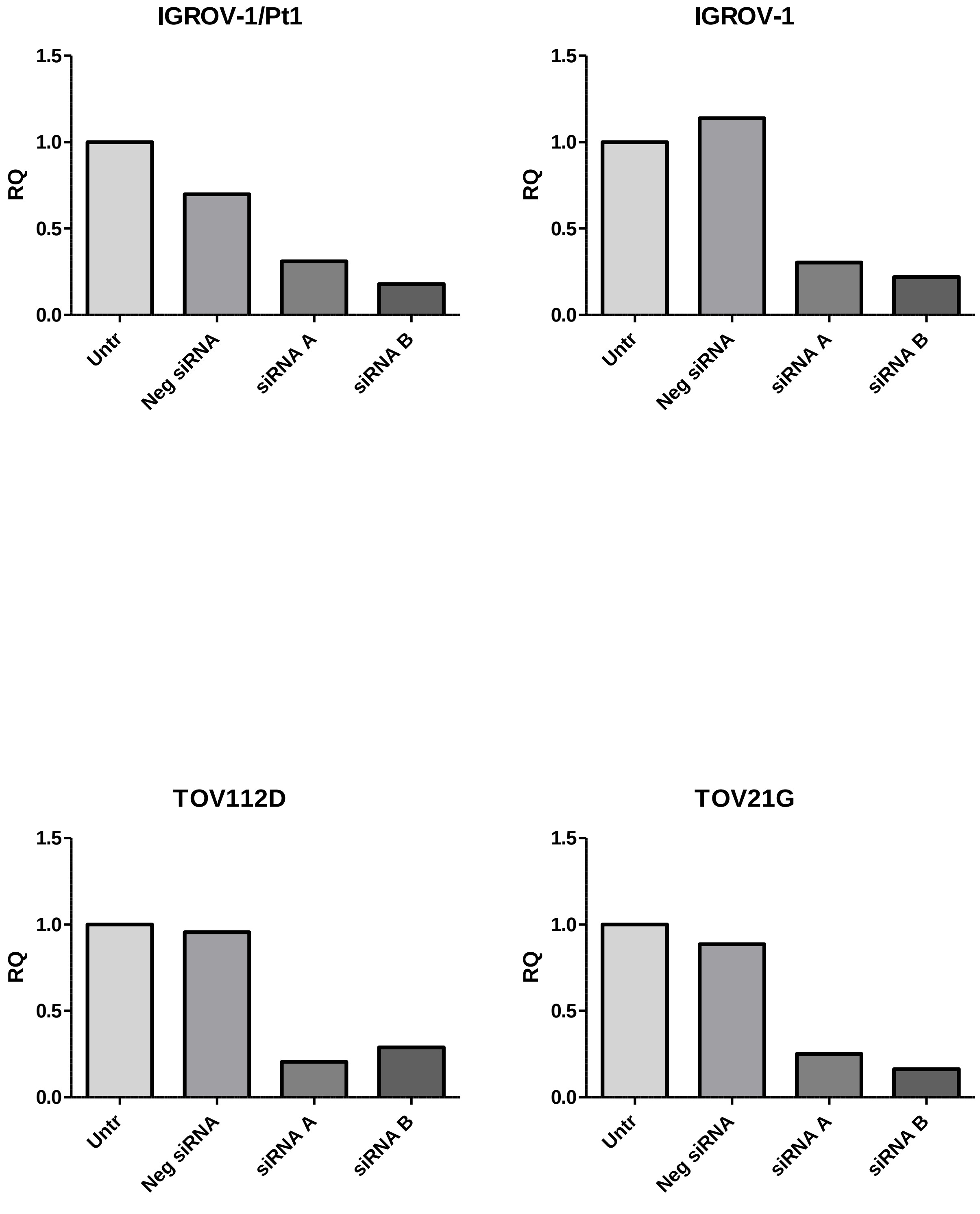

Figure S3: PSMC6 silencing in IGROV-1 cells and its effect on sensitivity to cisplatin by colony forming assay. a) Colony forming ability was evaluated in plastic dishes. Cells were harvested 48 h after siRNA transfection start and their colony forming ability was evaluated. **b)** Percentage of survival of IGROV-1 cells forming colonies in plastics after treatment with cisplatin at different concentrations. The IC_{50} values for each sperimental condition is reported.

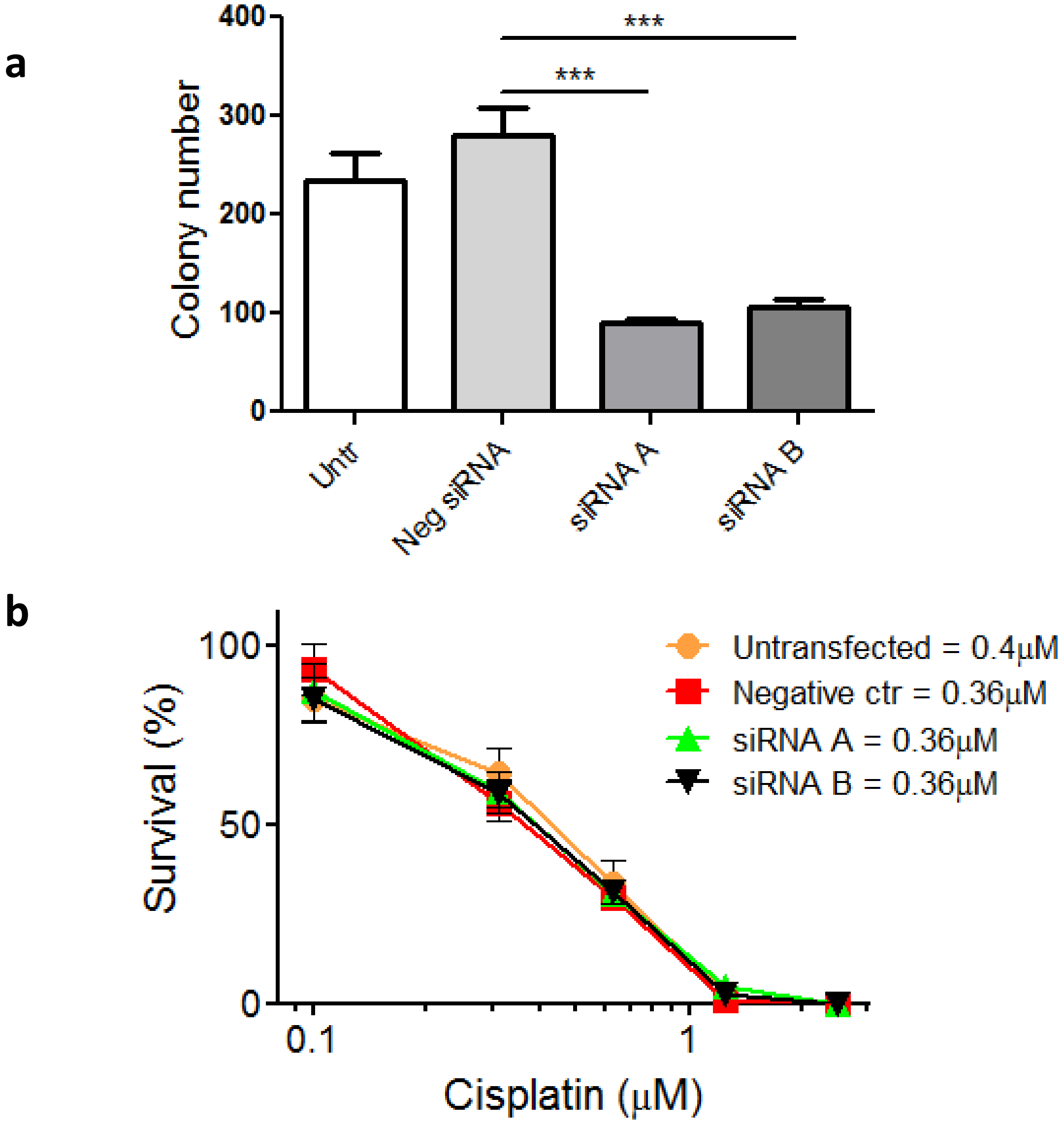

Figure S4. PSMC6 silencing in OSE-SV40 and FT240 cells and its effect on sensitivity to cisplatin by growth inhibition assay. Histograms represent the qRT-PCR of PSMC6 in silenced OSE-SV40 and FT240 cell lines. RNA of siRNA transfected cells was extracted and retrotranscribed into cDNA for RT-PCR for PSMC6 and GAPDH. The graphs represent the percentage of cell growth in growth inhibition of OSE-SV40 and FT240 cells after treatment with cisplatin at different concentrations. The IC_{50} values for each experimental condition is reported.

OSE-SV40

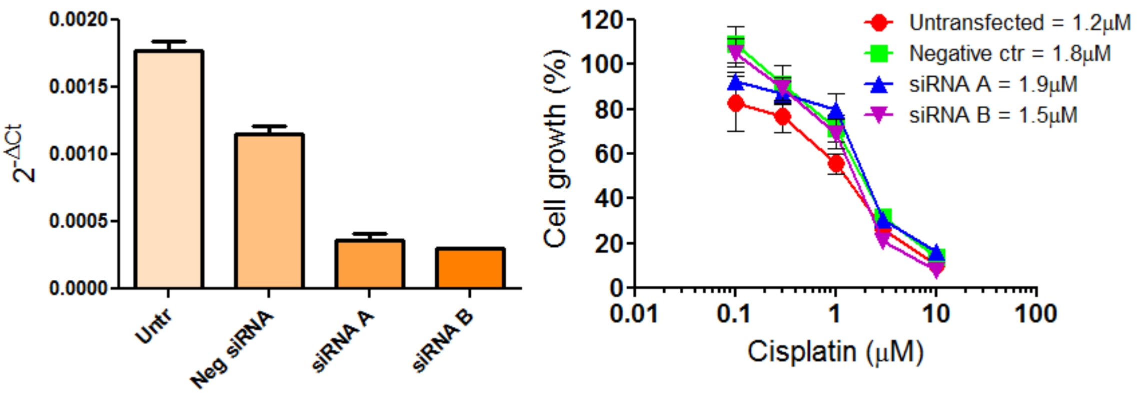

FT240

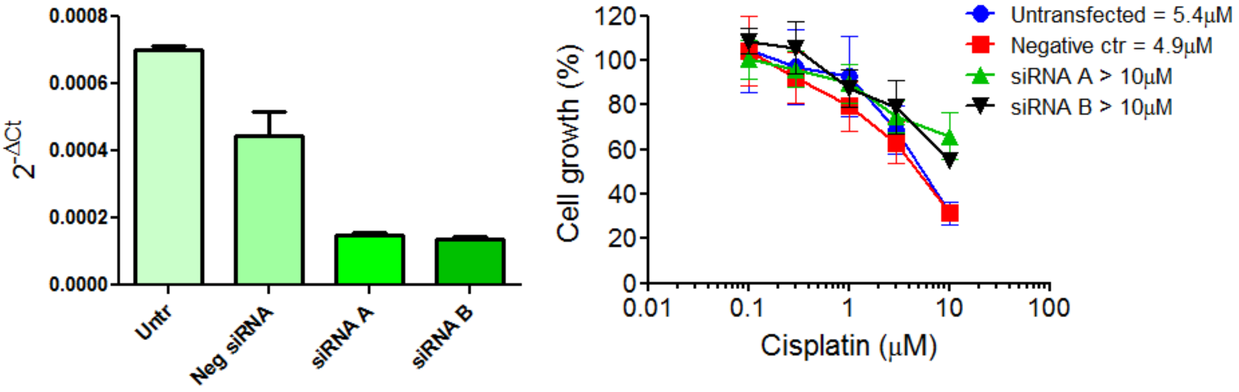

Figure S5. PSMC6 silencing in OVCAR-5, OVCAR-5/Pt and TOV21G/Pt and its effect on sensitivity to cisplatin by colony forming assay. Histograms represent the qRT-PCR of PSMC6 in silenced OVCAR-5, OVCAR-5/Pt and TOV21G/Pt cell lines. RNA of siRNA transfected cells was extracted and retrotranscribed into cDNA for RT-PCR for PSMC6 and GAPDH. The graphs represent the percentage of survival in clonogenic assay of OVCAR-5, OVCAR-5/Pt and TOV21G/Pt cells after treatment with cisplatin at different concentrations. The IC_{50} values for each sperimental condition is reported.

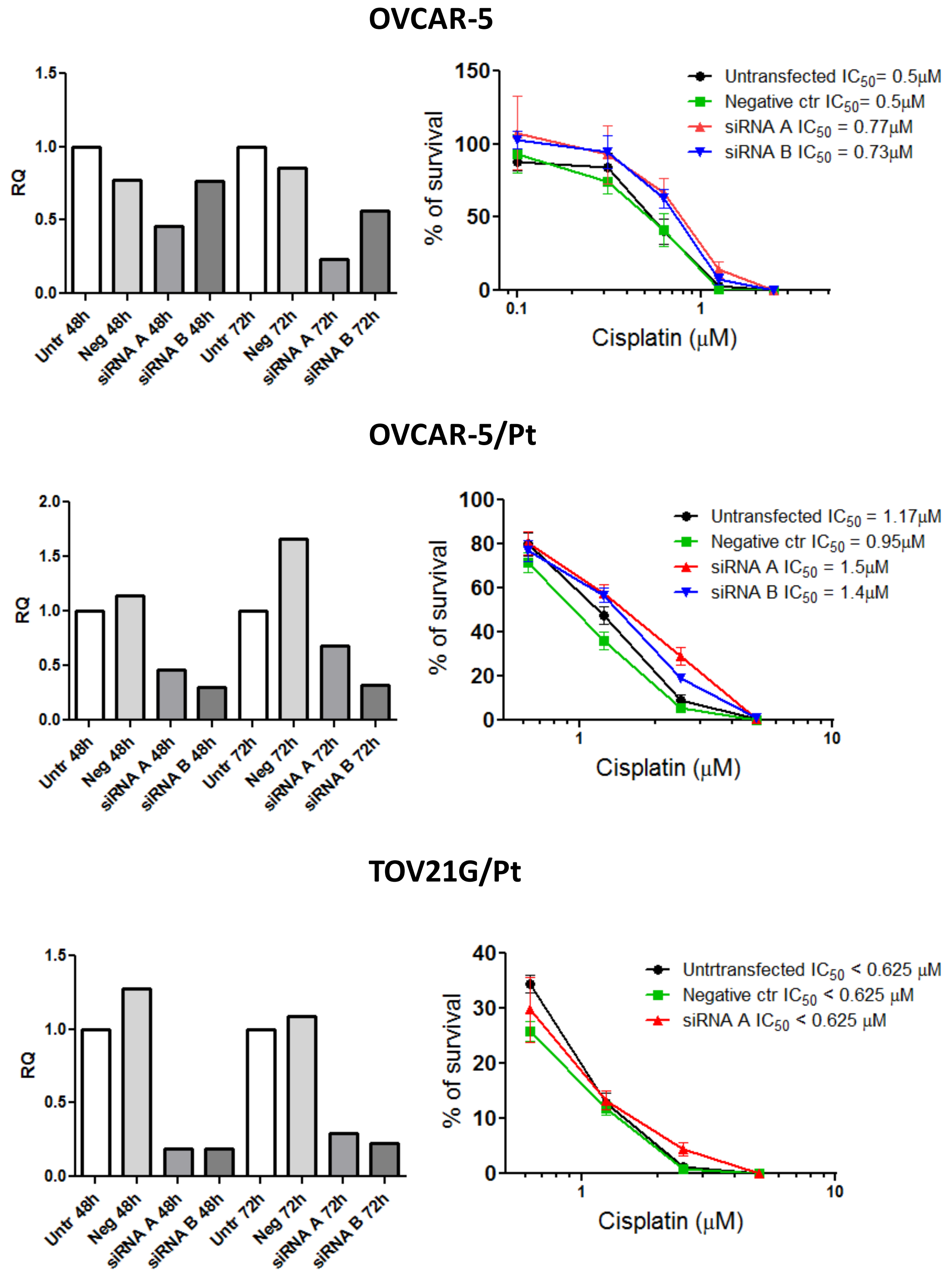

Figure S6. Analysis of ERK1/2 phosphorylation. Percentage of phospho-ERK1/2 over total ERK1/2 in IGROV-1 cell line after silencing with siRNAs. The extent of ERK1/2 phosphorylation has been measured by using an ELISA kit (Abcam)

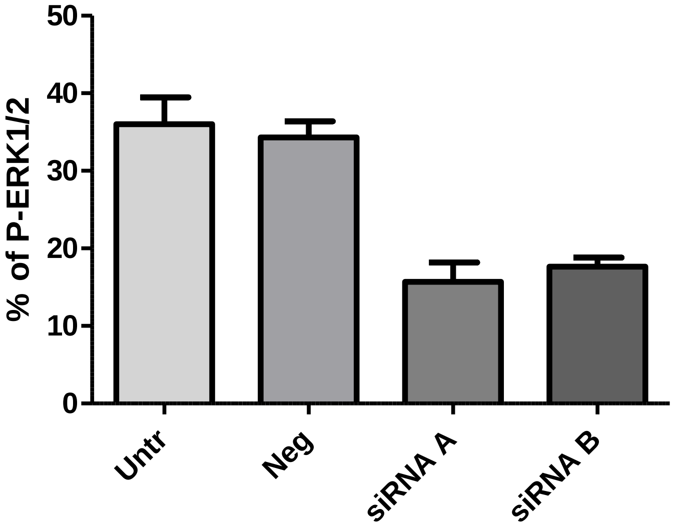

Figure S7. Human protein array and western blot analysis of selected proteins in PSMC6-silenced IGROV-1 and IGROV-1/Pt1 cells. Twenty for hours after seeding, cells were silenced with 100 nM control siRNA or siRNAs directed to PSMC6. Following 72 h, cells were harvested and lysed. Protein extracts were incubated overnight onto the array according to manufacturer's protocol. a), b) heatmap of the modulated proteins in IGROV-1/Pt1 (a) and IGROV-1cells (b). Western blot analysis of p21cip1/waf1 and cleaved Caspase-3 in IGROV-1/Pt1 and IGROV-1 cells (c). The band intensity was quantified by using the ImageQuant software. Western Blot quantification reported in **Table S6.**

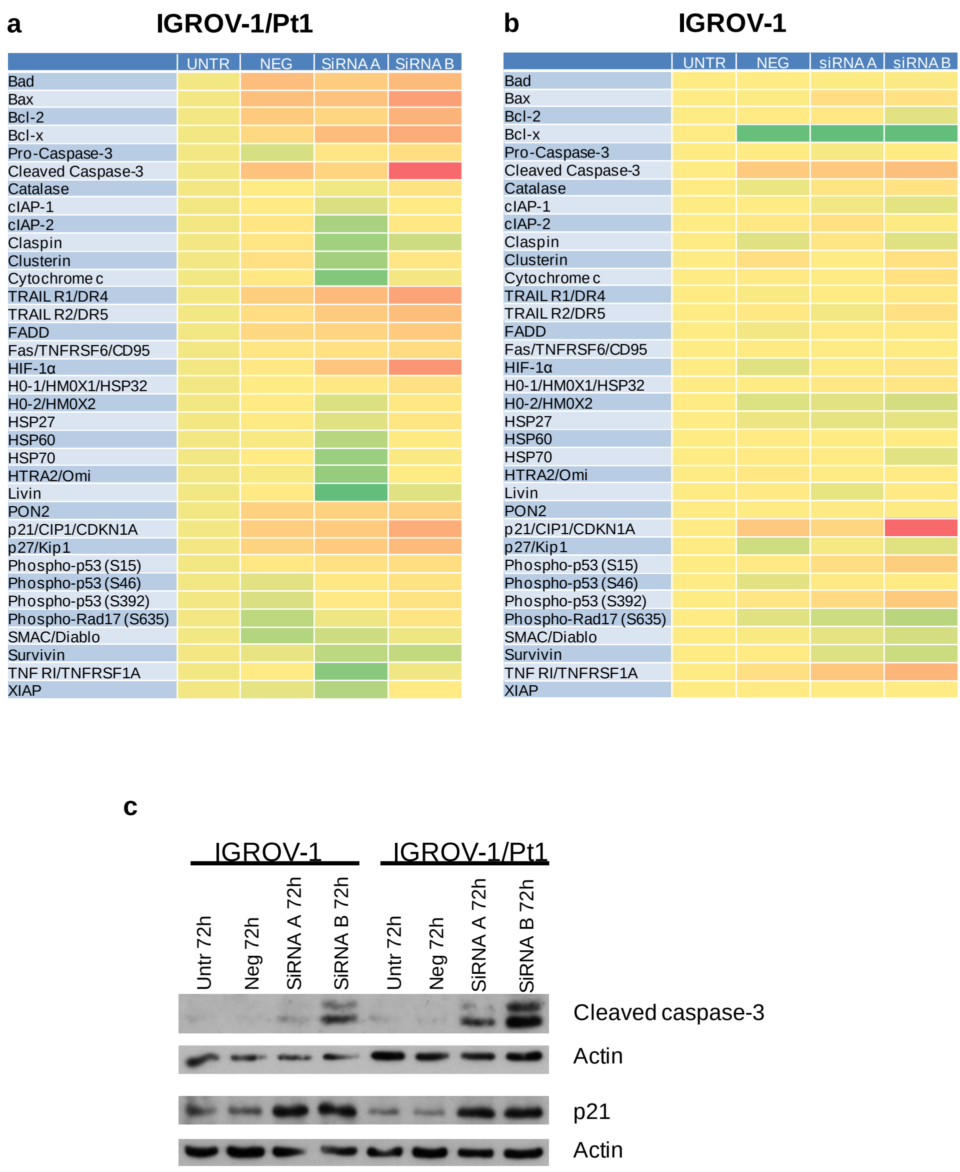

Figure S8. Human apoptosis antibody arrays of PSMC6-silenced IGROV-1 and IGROV-1/Pt1 cell lines. Untr, untransfected cells; Neg, negative control siRNA transfected cells; siRNA A, cells transfected with siRNA A; siRNA B, cells transfected with siRNA B

IGROV-1

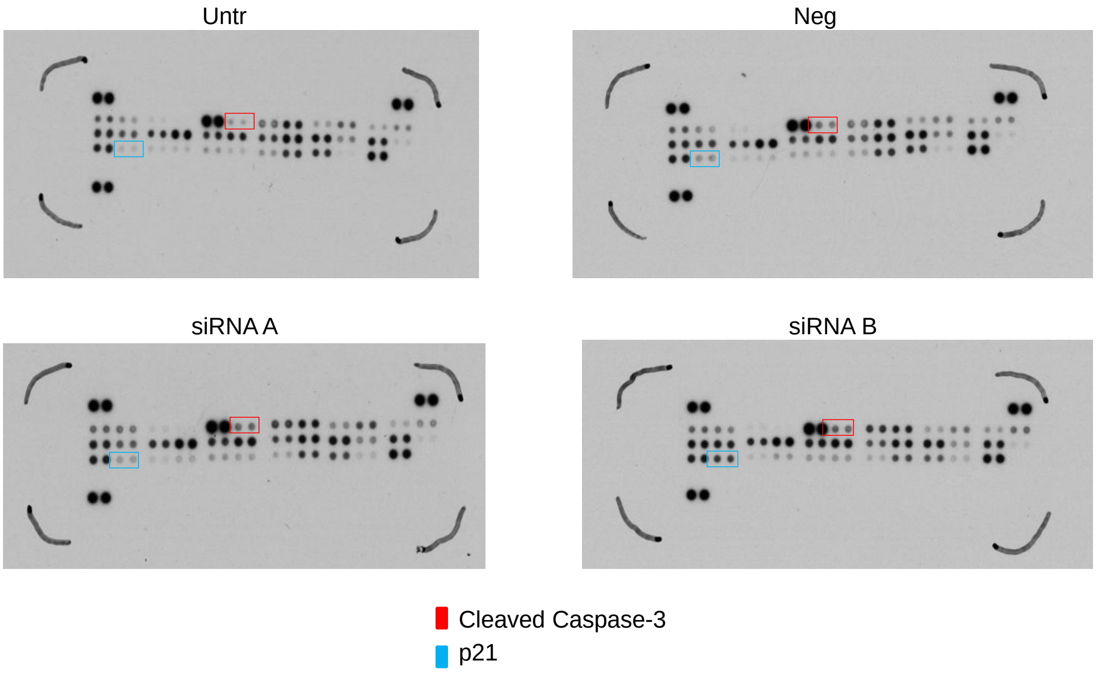

IGROV-1/Pt1

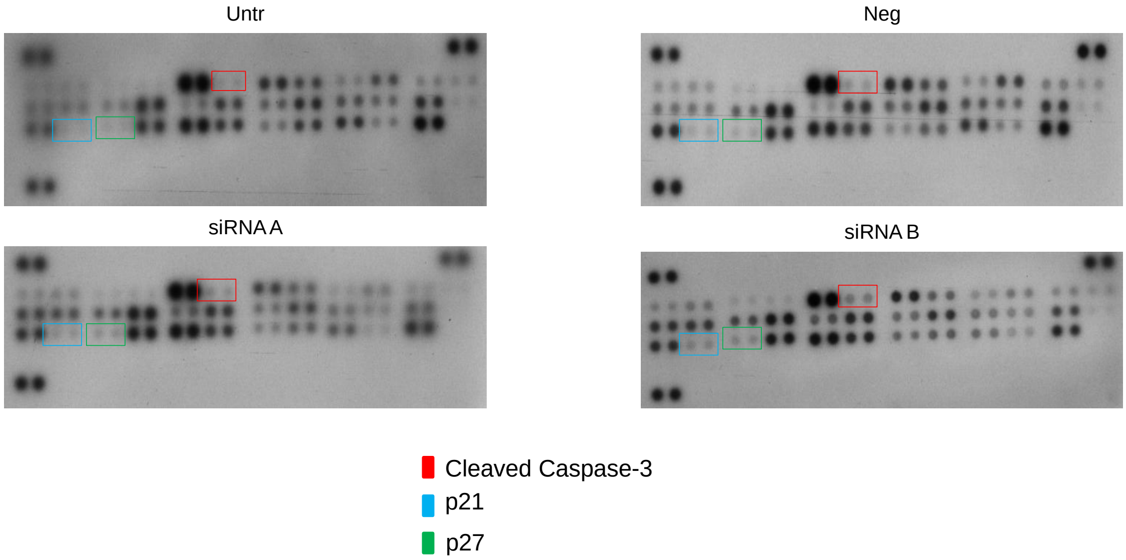

Figure S9. PSMC6 interaction network. The PSMC6 interaction network was built using STRING. The proteins functionally interacting with PSMC6 according to the results of the present study are reported. The thickness of the lines connecting the hubs represents the edge confidence.

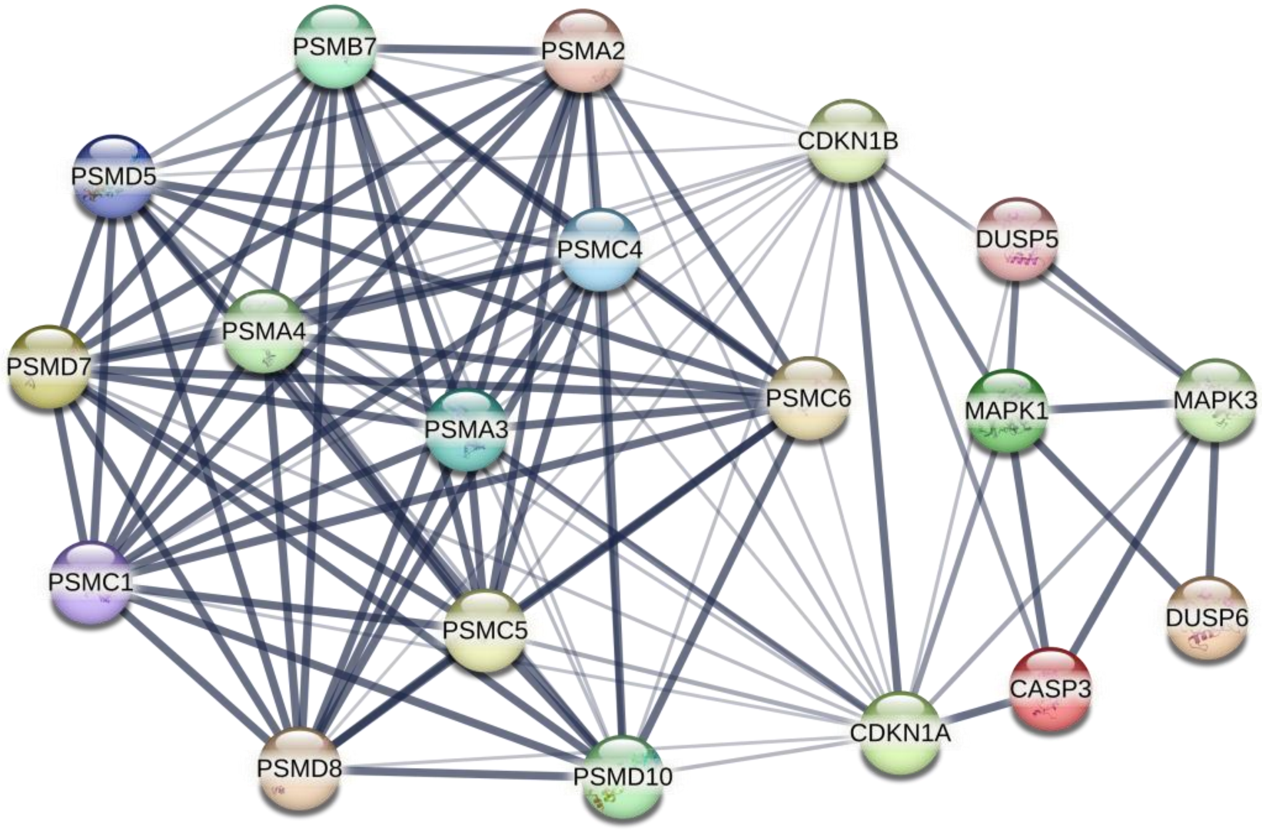

Figure S10. Sensitivity to proteasome inhibitors of IGROV-1/Pt1 (a) and IGROV-1 (b) cells following PSMC6 silencing. Cells were seeded and 24 h later silenced for 48 h with 100 nM siRNA A, siRNA B or Neg siRNA. Silenced cells were then harvested and seeded and 24 h later exposed to different concentrations of Bortezomib or bAP15 for 72h. Untr, untransfected cells; Neg siRNA, negative control siRNA transfected cells; siRNA A, cells transfected with siRNA A; siRNA B, cells transfected with siRNA B.

a

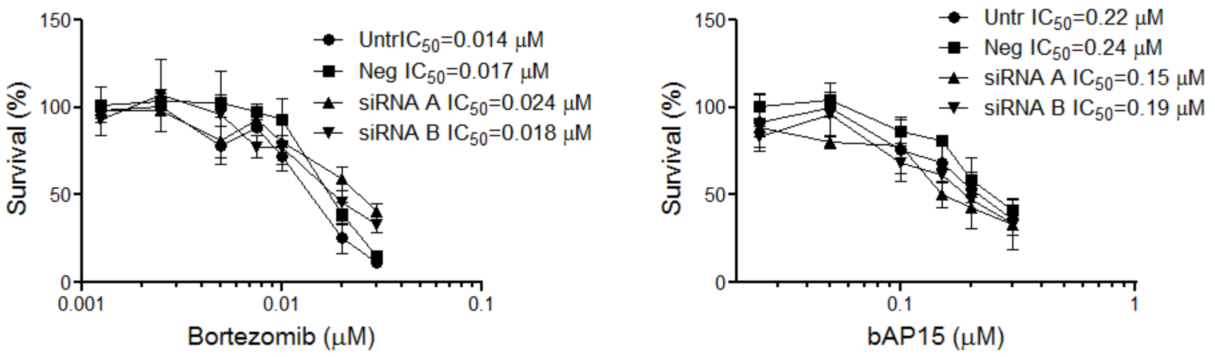

b

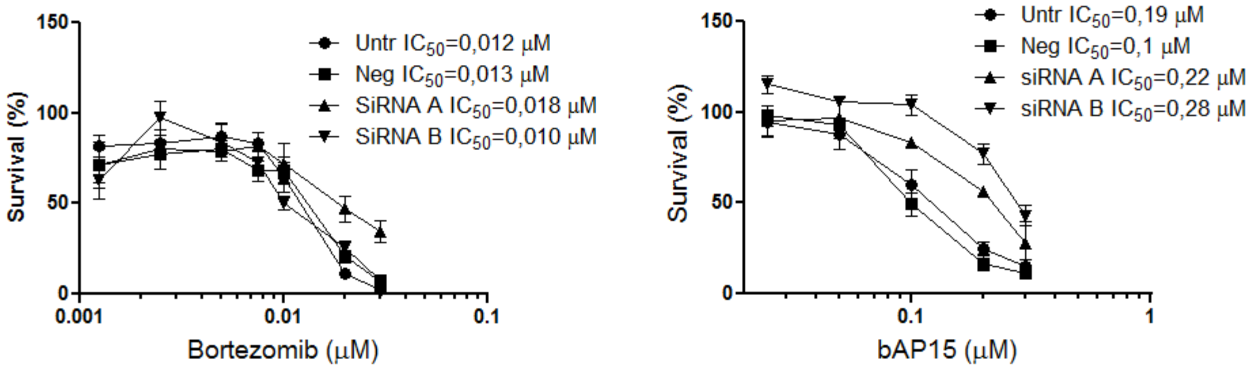

Figure S11. Phospho--ERK1/2 levels following PSMC6 silencing in serous OVCAR-5/Pt ovarian carcinoma cells. a) qRT-PCR of PSMC6 in silenced OVCAR-5/Pt cell lines. RNA of siRNA transfected cells was extracted and retrotranscribed into cDNA for RT-PCR for PSMC6 and GAPDH. **b)** Western blot assay of P-ERK1/2 and ERK in PSMC6 silenced OVCAR-5/Pt cells. Cells were harvested 72 h after transfection start for protein analysis. Cell lysates were analyzed by Western blotting. β-Tubulin represents control for loading.

a

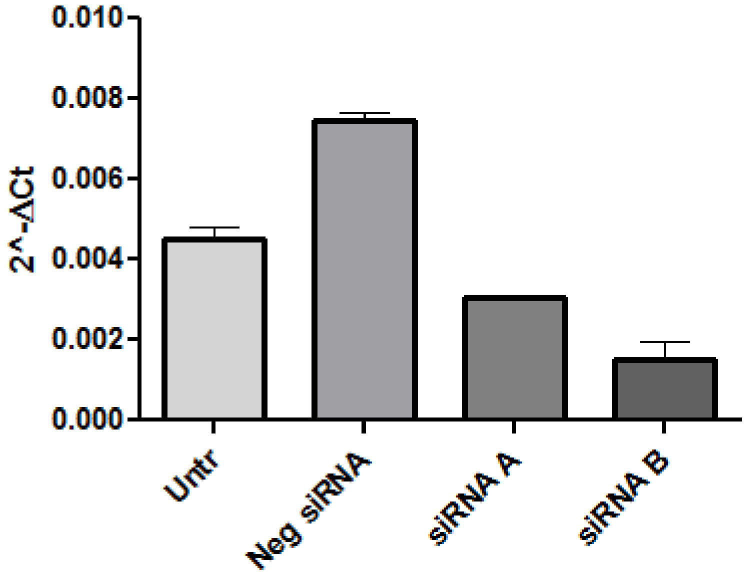

b

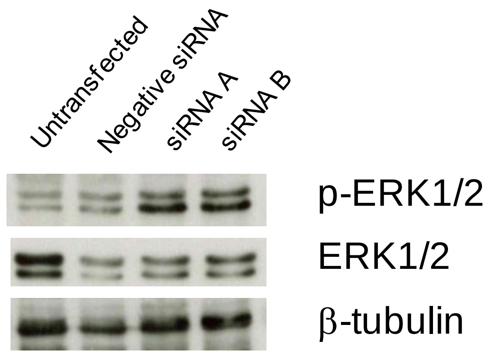

Supplement: Supplementary file 1 — Supplementary figures and tables. [file ijbsv21p2258s1.pdf]
